# Endometriosis in the era of precision medicine and impact on sexual and reproductive health across the lifespan and in diverse populations

**DOI:** 10.1096/fj.202300907

**Published:** 2023-08-29

**Authors:** Linda C. Giudice, Tomiko T. Oskotsky, Simileoluwa Falako, Jessica Opoku‐Anane, Marina Sirota

**Affiliations:** ^1^ UCSF Stanford Endometriosis Center for Innovation, Training, and Community Outreach (ENACT) University of California, San Francisco San Francisco California USA; ^2^ Center for Reproductive Sciences University of California, San Francisco San Francisco California USA; ^3^ Bakar Computational Health Sciences Institute University of California, San Francisco San Francisco California USA; ^4^ Columbia University Vagelos College of Physicians and Surgeons New York New York USA; ^5^ Division of Gynecologic Specialty Surgery Columbia University New York New York USA; ^6^ Department of Pediatrics University of California, San Francisco San Francisco California USA

**Keywords:** access, biomarkers, diagnosis, diversity, endometriosis, equity, health disparities, precision medicine, therapies

## Abstract

Endometriosis is a common estrogen‐dependent disorder wherein uterine lining tissue (endometrium) is found mainly in the pelvis where it causes inflammation, chronic pelvic pain, pain with intercourse and menses, and infertility. Recent evidence also supports a systemic inflammatory component that underlies associated co‐morbidities, e.g., migraines and cardiovascular and autoimmune diseases. Genetics and environment contribute significantly to disease risk, and with the explosion of omics technologies, underlying mechanisms of symptoms are increasingly being elucidated, although novel and effective therapeutics for pain and infertility have lagged behind these advances. Moreover, there are stark disparities in diagnosis, access to care, and treatment among persons of color and transgender/nonbinary identity, socioeconomically disadvantaged populations, and adolescents, and a disturbing low awareness among health care providers, policymakers, and the lay public about endometriosis, which, if left undiagnosed and under‐treated can lead to significant fibrosis, infertility, depression, and markedly diminished quality of life. This review summarizes endometriosis epidemiology, compelling evidence for its pathogenesis, mechanisms underlying its pathophysiology in the age of precision medicine, recent biomarker discovery, novel therapeutic approaches, and issues around reproductive justice for marginalized populations with this disorder spanning the past 100 years. As we enter the next revolution in health care and biomedical research, with rich molecular and clinical datasets, single‐cell omics, and population‐level data, endometriosis is well positioned to benefit from data‐driven research leveraging computational and artificial intelligence approaches integrating data and predicting disease risk, diagnosis, response to medical and surgical therapies, and prognosis for recurrence.

## Graphical abstract

Precision medicine is leading the way to resolving complex clinical challenges and disparities in diagnosing and treating many disorders and recently has been extended to endometriosis, a common, inflammatory disease with heterogenous symptoms and lesion subtypes causing chronic pain and infertility. Integrating complex metadata, high‐resolution omics data, imaging modalities, new surgical paradigms, and electronic health record mining are contributing to improved disease phenotyping, early diagnosis, novel drug discovery, personalized therapies, prognostic indicators, and risk of recurrence, across diverse populations.

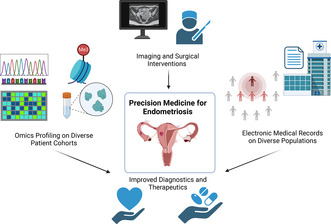

## 1 INTRODUCTION

Endometriosis is a common, estrogen‐dependent disease wherein tissue similar to the lining of the uterus (endometrium) exists outside its normal location, eliciting an inflammatory response, fibrosis, and pain.^1^, ^2^, ^3^ Although pelvic disease is most common, it can present, rarely, in extra‐pelvic sites (e.g., umbilicus, lymphatics, nerve roots, pleura of the lung, brain, pericardium).^4^ Recent research supports endometriosis as a systemic disorder transcending the reproductive organs and affecting mood, metabolism, autoimmune disorders, cancer risk, and the cardiovascular system.^5^, ^6^ It affects ~10% of reproductive‐age persons with a uterus, 60% with chronic pelvic pain, 80% with menstrual pain (dysmenorrhea), and 30%–50% of those with infertility.^2^, ^7^ Diagnosis is mainly surgical, as currently there are no disease biomarkers, and treatment is surgical removal of disease and/or minimizing estrogen action medically.^2^, ^7^ Endometriosis severely impacts quality of life^8^, ^9^, ^10^ and professional life,^11^ and health expenditures approximate $69B annually in the U.S.^12^, ^13^ Despite decades of research, with some progress in understanding the pathobiology of the disease, diagnosis and management are challenging, mainly because of its heterogeneous nature, multiple phenotypes, and associated systemic components.^3^

Despite its prevalence, there is limited understanding of endometriosis by health care professionals and the lay public, and cultural mores surrounding menstruation and pelvic pain in women, teens, and transgender men can disenfranchise those affected.^14^ While endometriosis is described as most prevalent in white women, race/ethnicity, socioeconomic, and gender factors may influence the ability to seek and access care for diagnosis and management,^15^, ^16^, ^17^ skewing prevalence data. Major unmet needs involving endometriosis include better understanding of the mechanisms underlying this multisystem disease, its onset and progression, response to treatments, and how genetic and environmental risks, racial and ethnic disparities, and socioeconomic status modulate these factors. Advanced molecular, clinical, and computational technologies and approaches to refine symptom tracking and quantification in real time and mining the rich resource of electronic health records are anticipated to complement multimodal, precision medicine approaches to disease diagnosis and management for all. Herein, we present an up‐to‐date assessment of epidemiology and pathogenesis and pathophysiology of endometriosis, an update on biomarker discovery and imaging approaches to disease diagnosis, and medical and surgical treatments in the context of disparities across populations and the lifespan.

## 2 MATERIALS AND METHODS

A comprehensive review of the literature was conducted from 1921 to 2023, using search engines and keywords including: adolescents, biomarkers, comorbidities, computational methods, diagnosis, drug repurposing, equity endometriosis, environment, epidemiology, epigenetics, ethnicity, genetics, health disparities, imaging, immune, immunology, infertility, inflammation, laparoscopy, menopause, menstruation, mHealth, mobile apps, pain, pathogenesis, pathophysiology, pregnancy, race, reproduction, robotic surgery, transcriptome, single cell, treatment, socioeconomic, surgery, medical, and systematic reviews. A narrative review was then constructed and focused on these keywords and concepts.

### 2.1 Epidemiology

Endometriosis has been found to affect approximately 10% of women, although prevalence estimates may vary considerably as studies differ methodologically in where they are conducted, the populations of individuals that are studied, and how endometriosis cases are defined. In general, estimates of the prevalence of endometriosis range from 0.8% to 11%, and endometriosis incidence from 4.2 to 35 per 10 000 women‐years.^18^ With respect to the reported likelihood of endometriosis diagnosis among different racial and ethnic groups, a meta‐analysis of at least 18 randomized control trials and observational studies found that, compared to White women, the likelihood of endometriosis diagnosis was less likely in Black women (OR: 0.49, 95% CI: 0.29–0.83) and Hispanic women (OR: 0.46, 95% CI: 0.14–1.50), and more likely in Asian women (OR: 1.63, 95% CI: 1.03–2.58).^15^ However, true prevalence rates are limited by who has access to laparoscopic/robotic surgery for diagnosis.^16^ In a retrospective analysis of transgender men who underwent laparoscopic hysterectomy for gender affirmation, endometriosis was found among 26.9% (18/67 patients), a higher rate than previous report of 16.9% (38/225 patients) for women who underwent laparoscopic ovarian drilling and of 11.8% (55/465 patients) for women who underwent laparoscopic tubal sterilization.^19^, ^20^, ^21^ While endometriosis predominantly affects women of reproductive age, it can also affect those who are pre‐menarchal or post‐menopausal.^22^, ^23^, ^24^ In a retrospective study of 42 079 women with histologically confirmed endometriosis, 80.36% (33 814 patients) were 0–45 years of age including 0.05% (23 patients) who were under 15 years of age, 17.09% (7191 patients) were 45–55 years of age, and 2.55% (1074 patients) were over age 55 years.^22^

Determining the prevalence of endometriosis is challenging because individuals can be asymptomatic or have varied and non‐specific symptoms, and definitive diagnosis generally requires surgery.^25^ Studies identifying endometriosis cases with patient self‐report generally report higher prevalence and incidence estimates relative to studies using electronic medical record (EMR) data, particularly those conducted outside the United States.^18^ Moreover, biases such as prevailing beliefs about whom endometriosis affects (e.g., high‐achieving, affluent women with private health insurance and who delay childbearing) as well as disparities in health care access can affect the likelihood of individuals being diagnosed with this disease.^16^

As for endometriosis phenome, increased risk of disease includes family history, nulliparity, prolonged exposure to endogenous estrogen (e.g., early menarche (≤ age 11 years) or late menopause), shorter menstrual cycles (i.e., <27 days), heavy menstrual bleeding, obstruction of menstrual outflow, exposure in utero to diethylstilbestrol, adult environmental exposures to polychlorinated biphenyl (PCB) or dioxin, taller height, lower body mass index, and alcohol or caffeine intake.^26^, ^27^, ^28^, ^29^, ^30^ Factors associated with a decreased risk of endometriosis include higher parity, extended lactation intervals, late menarche (>age 14 years), and exercise.^26^, ^29^

### 2.2 Pathogenesis

#### 2.2.1 Theories of disease origin

**FIGURE 1:**
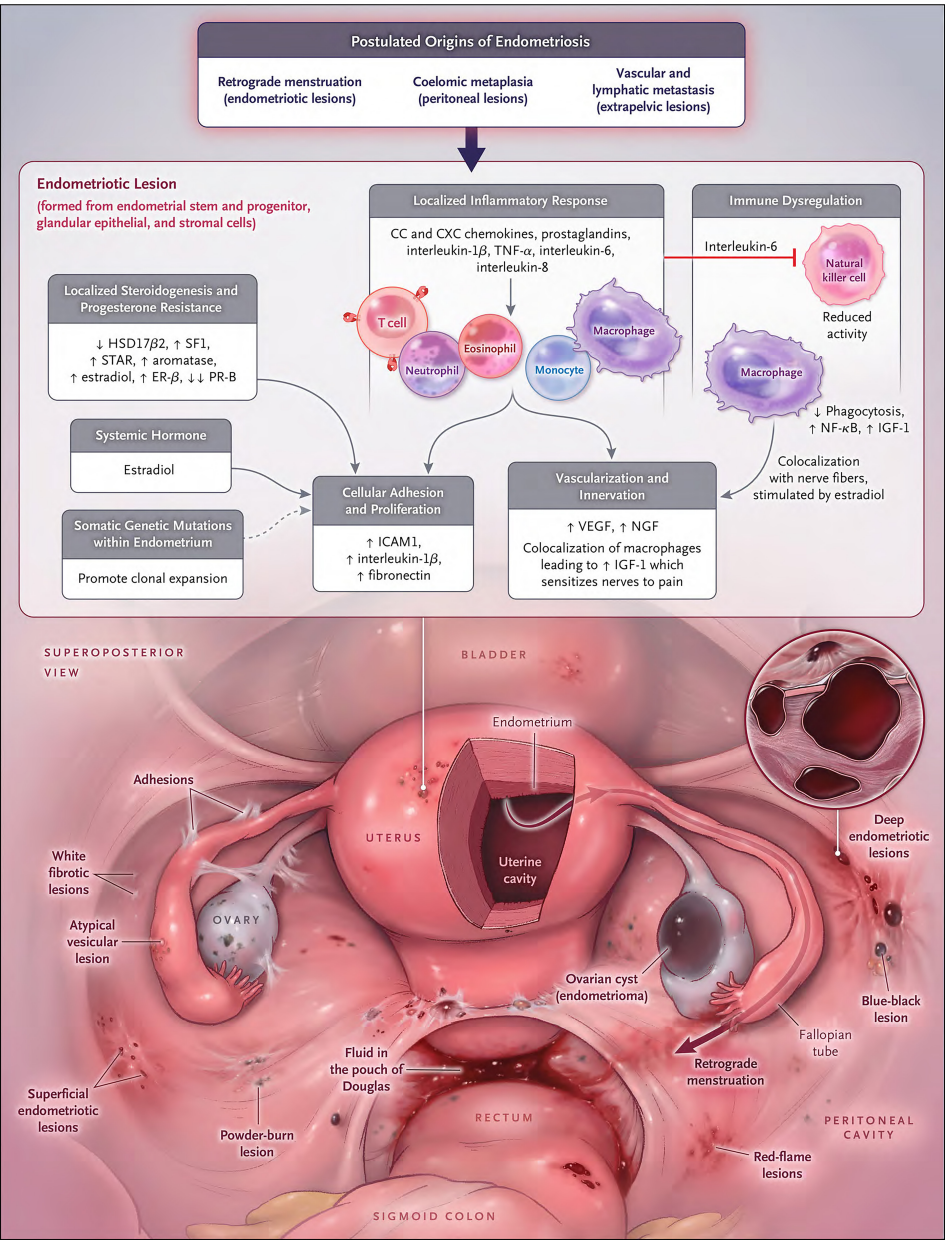
Model of pelvic endometriosis pathogenesis and pathophysiology. Origins of endometriotic lesions include transplantation of endometrial tissue fragments and cells via retrograde menstruation and coelomic metaplasia of the peritoneal mesothelium; and stem and progenitor cell differentiation. Vascular and lymphatic metastasis likely give rise to disease in extra‐pelvic sites. When superficial and deeply invasive lesions develop, they are maintained via molecular mechanisms that promote cellular adhesion cell proliferation, a localized inflammatory response, immune dysregulation, neoneuroangiogenesis, and systemic and localized steroidogenesis. Dashed arrow shows postulated effects. ER, estrogen receptor; HSD17β2, 17β‐hydroxysteroid dehydrogenase 2; ICAM, intercellular adhesion molecule; IGF, insulin‐like growth factor; NF‐κB, nuclear factor κB; NGF, nerve growth factor; PR, progesterone receptor; SF1, steroidogenic factor; STAR, steroidogenic acute regulatory protein; TNF, tumor necrosis factor; VEGF, vascular endothelial growth factor. From Ref. [3]: Zondervan KT, Becker CM, Missmer SA. Endometriosis. N Engl J Med. 2020;382(13):1244–1256. 10.1056/NEJMra1810764, with permission.

**FIGURE 2:**
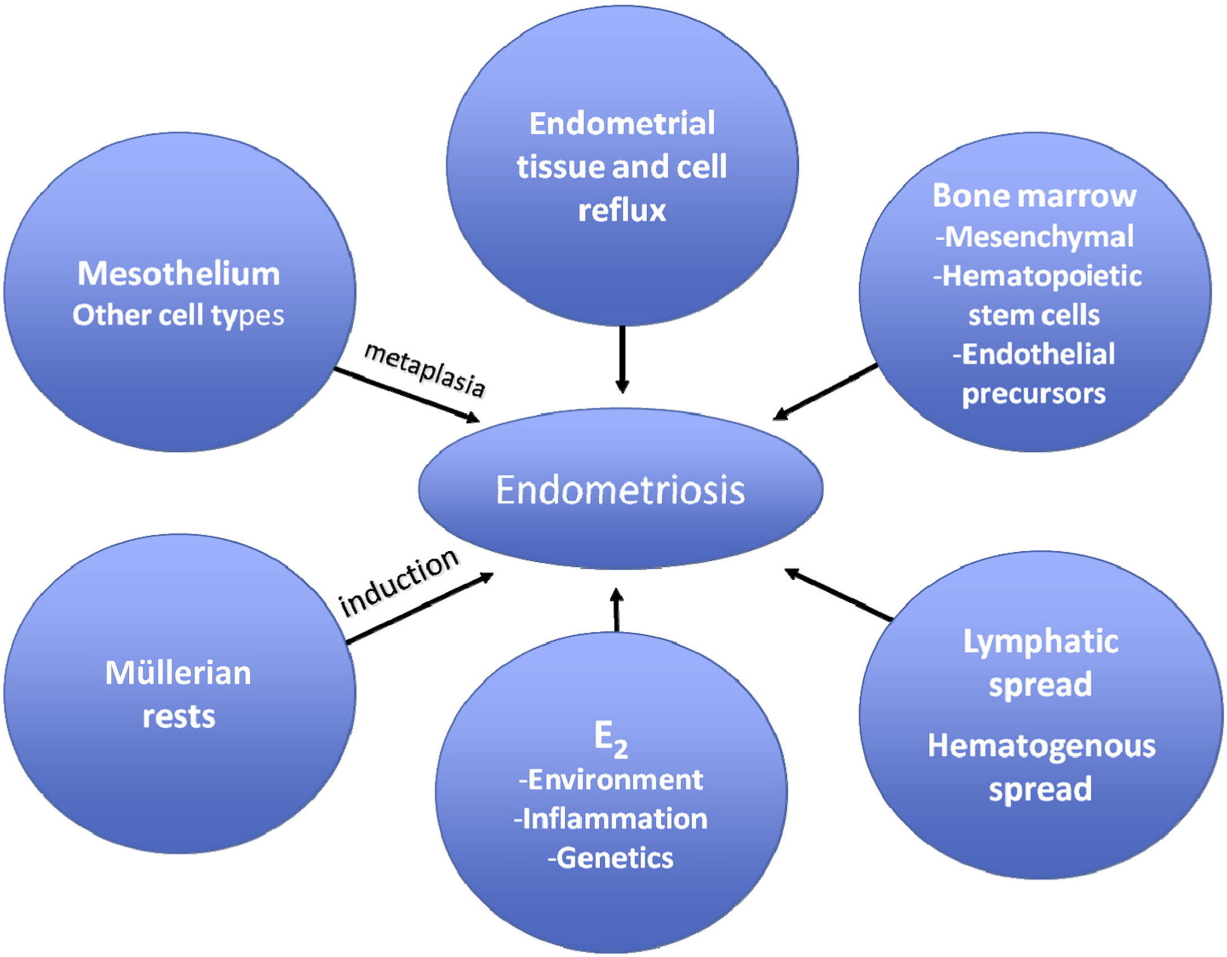
Theories regarding endometriosis pathogenesis. Multiple theories of endometriosis prevail and shown here include: retrograde deposition of menstrual blood and tissue into the pelvis, lymphatic and/or hematogenous spread to distant sites, bone marrow mesenchymal and hematopoietic stem cell migration to the pelvic cavity and/or endometrium, endothelial cell transformation to endometrial cells, induction of disease by remnant Mullerian rests, metaplasia of mesothelium by uncertain triggers, and estrogenic endocrine disrupters, excessive estradiol (E2), inflammation accompanying and sustaining disease, and a genetic predisposition to disease. From Ref. [4]: Burney RO, Giudice LC. Pathogenesis and pathophysiology of endometriosis. Fertility and Sterility. 2012;98(3):511–519. 10.1016/j.fertnstert.2012.06.029, with permission.

There are three main types of endometriosis based on histopathology and anatomic location.^4^, ^31^, ^32^, ^33^ These include ovarian endometrioma cysts lined by endometrial (not ovarian) cells, superficial disease that penetrates <5 mm into sub‐serosal peritoneal soft tissue or visceral organs, and deep infiltrating disease that extends >5 mm into the muscular layer of the intestine, bladder wall, diaphragm, rectovaginal septum, and other areas) (Figure 1).^3^ The most commonly accepted theory of endometriosis pathogenesis, proposed by Sampson in 1927,^34^ is that endometrial cells and tissue fragments, refluxed through the fallopian tubes during menses, arrive on the pelvic peritoneum, visceral tissues, and the ovarian surface, set up an inflammatory reaction and promote disease lesion establishment and invasion. Sampson also proposed that menstrual dissemination into the venous circulation could promote “embolic” disease at extra‐pelvic sites^35^ and that ovarian endometriomas derive from invagination of menstrual debris into the ovarian cortex.^36^ Over the next ~100 years, several lines of experimental evidence, using animal models, human tissues, cells, and fluids and multi‐omic approaches, have supported retrograde menstruation, hematogenous spread, and other pathogenic mechanisms and have provided unique insights into endometrial cell types involved in disease establishment, survival, and growth (Figure 2, ^4^). These mechanisms include resident endometrial stem/progenitor cells shed at menses or with neonatal uterine bleeding and implanted in the pelvic tissues giving rise to rare pre‐menarchal disease and to adolescent and adult disease^37^; hematogenous spread of bone marrow‐derived mesenchymal stem cells, hematopoietic stem cells, and endothelial precursors to the endometrium, which when shed at menses into the pelvis, result in lesion formation^38^; coelomic metaplasia of mesothelial cells initiated by menstrual blood or other “irritants”; induction of Mullerian rests by uncertain triggers giving rise to deep infiltrating disease in select anatomic sites; and cell transformation/induction *in utero* and across the life course by estrogenic endocrine‐disrupting chemicals.^4^, ^33^, ^37^

#### 2.2.2 Somatic mutations supporting eutopic endometrium giving rise to endometriosis

Next‐generation whole genome sequencing has demonstrated that endometrial epithelial cells carry cancer driver mutations that are shared with ovarian endometrioma, and other mutations that are shared with surface and deep infiltrating disease, without cancer,^33^, ^39^, ^40^ strongly supporting the retrograde menstruation/implantation theory. Moreover, within lesions, the epithelial population is clonal, whereas the stromal cells are not, suggesting that lesion formation involves a single epithelial cell (or progenitor that clonally differentiates into epithelium) that recruits multiple, independent polyclonal stromal cells.^33^, ^41^ Notably, clonal expansion of epithelial cells with cancer‐associated mutations has been proposed to lead to the development of endometriomas.^42^ Further studies are needed to confirm these observations and predictions, and the possible relationship of cancer driver mutations with ovarian endometrioma‐related cancer risk, as well as exploring the applicability of these findings in diverse populations.

#### 2.2.3 Genetic susceptibility to endometriosis

As nearly all individuals with a uterus have some degree of retrograde menstruation,^43^ the question arises as to why most do not develop endometriosis. Genetics and the environment appear to be at play in this regard, although a single gene mutation and specific environmental triggers, times of exposure, and periods of vulnerability have not been identified specifically. Recent technological advances and reduced cost of genotyping and sequencing have allowed extensive profiling of genetic signals in the context of endometriosis. A brief overview of genetic contributions is presented herein, which have recently been reviewed in detail.^3^, ^27^, ^30^, ^44^

Diagnosis among first‐degree relatives of patients with endometriosis is 2‐ to15‐fold higher than for relatives of unaffected individuals,^7^, ^30^ and large twin studies reveal a heritable component of ~ 50% (0.51,^45^ 0.47^46^), with ~26% estimated due to common genetic variation.

Genetic linkage and candidate gene approaches have not confirmed single, highly penetrant polymorphisms, which is not unexpected given that endometriosis is a multifactorial, complex trait.^7^, ^30^ However, genome‐wide association studies (GWAS), using high‐throughput genotyping technologies and advanced bioinformatics analyses (https://www.genome.gov/GWAStudies; http://www.ebi.ac.uk/gwas), have transformed understanding genetic contributions to complex diseases, identified sequence variants associated with disease phenotypes, and are beginning to translate these findings to co‐morbid conditions.^47^ A recent GWAS meta‐analysis of 60 674 cases and 701 926 controls of European and East Asian descent identified 42 genome‐wide significant loci with effect sizes greatest for stage III/IV disease (staging described below), driven mainly by ovarian endometriomas.^48^ Some loci involved sex steroid hormone pathways and metabolism (*ESR1, GREB1, FSHB)* and Wnt signaling, which could contribute to the known estrogen‐driven etiology and pathophysiology of endometriosis, although precise genetic risk contributions to mechanisms underlying disease establishment and/or progression remain to be determined. Notably, some loci regulated expression or methylation of genes in endometrium and blood associated with pathophysiologic processes of pain perception and maintenance, and significant genetic correlations were found between endometriosis and 11 pain conditions, including migraine, back pain, multi‐site chronic pain, and inflammatory conditions (asthma and osteoarthritis).^48^ A recent GWAS of five gynecologic diseases and cross‐trait analysis in Japanese women found strong genetic correlations between endometriosis and ovarian cancer, ovarian and endometrial cancer, and uterine fibroids and ovarian cancer,^49^ suggesting common susceptibilities for their development and/or pathophysiology. With regard to uterine fibroids, a common comorbidity with endometriosis, a recent GWAS meta‐analysis^50^ of 35 474 cases with uterine fibroids and 267 505 female controls of white European ancestry found four of 29 loci significantly associated with fibroids that overlapped estrogen and progesterone signaling pathways (see above) in endometriosis patients. This study did not confirm five loci previously identified in African American persons with uterine fibroids,^51^ possibly due to ancestral differences or phenotypic definitions.^50^

Targeted investigations of genetically regulated mechanisms shared between endometriosis and other conditions are anticipated to give insights into novel therapies for endometriosis and related pain and other comorbidities. However, GWAS for endometriosis risk to date focus mainly on European and East Asian populations.^30^, ^52^ Therefore, there is a need for trans‐ethnic GWAS for endometriosis, as has been done, e.g., for uterine fibroids,^53^ to allow trans‐ethnic signal fine‐mapping, characterize effect sizes of variants in different ethnic groups, and identify novel variants among non‐European ancestry populations.

### 2.3 Pathophysiology

#### 2.3.1 Role of estrogen and progesterone in endometriosis

**FIGURE 3:**
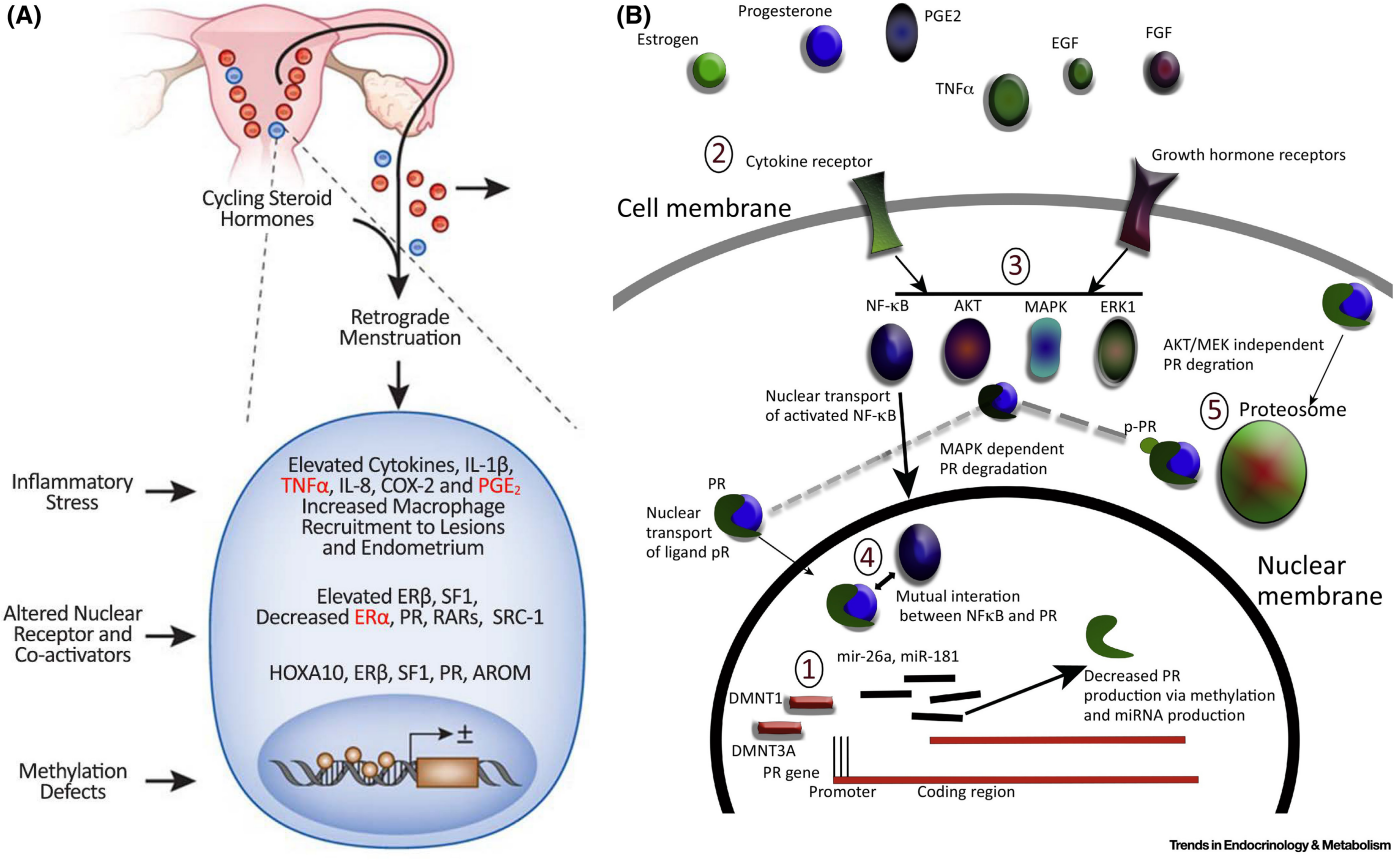
Models of inflammation and aberrant steroid hormone signaling in the pathophysiology of endometriosis. A. Inflammation, enhanced estrogen signaling. Panel A depicts retrograde menstruation resulting in inflammatory stress and associated cytokines, inflammatory mediators, and macrophage recruitment to lesions, altered nuclear receptors and co‐activators, and methylation defects resulting in altered gene transcription and enhanced estrogen signaling. B. Mechanisms of disrupted progesterone signaling. Mechanisms include (indicated by numbers in ovals): (1) Inhibition of PR transcription by increased PG promotor DNAme and altered DMNT1 and DMNT3A and B; (2) stimulation of cell membrane receptors by hormones, cytokines, and growth factors; (3) activation of AKT, ERK1, MAPK that suppress PR activity via increased phosphorylation and degradation via proteasome pathways; (4) Inflammation stimulates NF‐kB activation and NF‐kB interacts with PR, leading to reduced PR expression; and (5) Non‐AKT, MEK, ligand binding‐induced degradation can also regulate PR protein levels. AKT, akt serine/threonine protein kinase; AROM, aromatase; COX‐2, cyclooxygenase 2; DMNT, DNA methyl transferase; DNAme, DNA methylation, EGF, epidermal growth factor; E_2_, estradiol; ERβ, estrogen receptor beta; ERK1, extracellular signal‐regulated kinase; FGF, fibroblast growth factor; Il1b, interleukin 1 beta; MAPK, mitogen‐activated protein kinase; mir, microRNA; NF‐kB, nuclear factor kappa‐light‐chain‐enhancer of activated B cells; PGE_2_, prostaglandin E2; PR, progesterone receptor; pPR, phosphorylated PR; RARs, retinoic acid receptors; SF1, steroidogenic factor 1; SRC‐1, steroid receptor coactivator‐1; TNF‐α, tumor necrosis factor alpha. Adapted from Ref. [54]: Dyson MT, Bulun SE. Cutting SRC‐1 down to size in endometriosis. Nat Med. 2012;18(7):1016–1018. doi:10.1038/nm.2855 (Panel A), with permission, and from Ref. [55]: McKinnon B, Mueller M, Montgomery G. Progesterone resistance in endometriosis: an acquired property? Trends Endocrinol Metab. 2018;29(8):535–548. 10.1016/j.tem.2018.05.006 (Panel B), with permission.

Central to endometriosis pathophysiology are enhanced estrogen and disrupted progesterone (P_4_) signaling and inflammation (Figure 3). These two processes are interrelated and largely contribute to the pain and infertility in affected individuals. Retrograde menstruation leads to peritoneal inflammation, with elevated cytokines, IL‐1β, TNFα, IL‐8, COX‐2, and PGE_2_ and increased macrophage recruitment to lesions and endometrium (Figure 3A).^54^ Notable is the prominent role of activated and dysfunctional circulating, peritoneal, and tissue‐resident myeloid lineage cells that not only are pro‐inflammatory but also fail to clear lesions in ectopic sites.^56^, ^57^, ^58^, ^59^, ^60^ There are also altered nuclear receptors and co‐activators in lesions and corresponding eutopic endometrium, including elevated Erβ, SF1, and decreased Erα, PR, RARs, and SRC‐1. Some mediators are regulated by methylation defects, including hypermethylation of PR and HoxA10 and hypomethylation of Erβ, SF1, and aromatase (AROM).^61^ Thus, the imbalance of increased estradiol (E_2_) synthesis and action and decreased progesterone action (see below) largely drive the disease pathophysiology and are the basis of medical therapies for endometriosis‐associated pain.

In addition to enhanced estrogen signaling, aberrancies in P_4_ signaling are well documented.^61^, ^62^, ^63^ Mechanisms underlying abnormal P_4_ signaling (Figure 3B)^55^ include transcriptional regulation of PR expression with increased DNA‐methylation at the promoter and first exon and altered DMNT1,3A/B; post‐transcriptional over‐expression miR‐26a and miR‐181, which block E_2_‐dependent PRA and PRB in breast cancer cell lines; cytokines, hormones, and growth factors stimulating cognate receptors, activating AKT, ERK1, and MAPK pathways that suppress PR activity by increased phosphorylation and degradation via proteasome pathways; and inflammatory mediators (TNFa, EGF, FGF) stimulating NFkB activation that has mutual interaction with PR leading to reduced PR expression.

The environment is a possible contributor to the above epigenetic changes, and extensive evidence^64^, ^65^ supports a role for endocrine disrupting chemicals (EDCs) resulting in epigenetic modifications in the genome, including aberrant DNA methylation, histone modifications, and altered non‐coding RNAs. Whether exposures to EDCs directly result in some of the modifications noted above relevant to endometriosis pathophysiology is uncertain. However, recent systematic reviews and meta‐analyses of the epidemiologic literature conclude higher risk of endometriosis diagnosis with higher exposures to common EDCs, including dioxins [OR: 1.56 (95% CI: 1.14–2.39)], polychlorinated biphenyls (PCBs) [OR: 1.70 (95% CI: 1.20–2.39); 1.58 (95% CI: 1.18–2.12)], organochlorine pesticides (OCPs) [OR: 1.97 (95% CI: 1.25; 3.13); 1.40 (95% CI: 1.02–1.92)], and phthalate esters, as DEHP [OR: 1.42 (95% CI: 1.19–1.70)].^66^, ^67^ Table 1 shows persistent and non‐persistent EDCs, their sources, and associations with endometriosis (and other gynecologic disorders).^68^ There are many challenges in conducting these types of studies including uncertain exposure timing and duration during development and/or across the life course; dynamic exposures simultaneously to mixtures not solely individual EDCs; uncertainty of diagnosis of a common disorder without disease biomarkers; heterogeneity of the disease; confounders of age, BMI, parity, breastfeeding, cigarette smoking, alcohol, medications, co‐morbidities; definition of the control group; choice of tissue or fluid to quantify chemicals (especially lipophilic); choice of study design (case‐control or cohort); and outcome ascertainment (pain, infertility).^69^ Also, the majority of the studies to date have been carried out in white patients. Despite these caveats and the small effect sizes observed, the data overall suggest that specific EDCs or their metabolites may promote endometriosis, and, notably, those most vulnerable to EDC exposures are socioeconomically disadvantaged minority populations.^70^ Further research on EDC exposure and endometriosis risk is warranted, and mitigating these risks will be important to improve the health of persons with endometriosis and perhaps to prevent the disorder and its myriad of phenotypes and co‐morbidities—especially with a focus on vulnerable populations.

**TABLE 1:** Reproductive health impacts of selected endocrine‐disrupting chemicals (EDCs).

Type of endocrine‐disrupting chemicals	Endocrine‐disrupting chemicals	Sources of exposure	Industrial benefits	Potential gynecologic health risks
Persistent EDCs	Dioxins	Combustion, waste incineration, volcanic eruptions, forest fires	N/A	Endometriosis, Adenomyosis, Reproductive cancers
	Polychlorinated Biphenyls (PCBs)	Electrical transformers, microscope immersion oils, pesticides, carbonless copy paper	Electrical insulating compounds	Endometriosis, Adenomyosis, uterine fibroids
Non‐persistent EDCs	Bisphenol A (BPA)/Bisphenol S (BPS)	Children's toys, water bottles, canned food liners, dental sealants, receipt coatings	Plasticizer and epoxy resins	Endometriosis, uterine fibroids, polycystic ovarian syndrome, adenomyosis
	Phthalates	Cosmetics, medical equipment, medications, paints, adhesives, personal care products	Plasticizers, solvents, and stabilizers	Endometriosis, uterine fibroids, adenomyosis
	Parabens	Cosmetics, pharmaceutical products	Preservatives	Endometriosis, uterine fibroids
	Triclosan (TCS)	Hand sanitizers, mouthwash, toothpaste	Antimicrobial properties	Polycystic ovarian syndrome

*Note*: Stephens et al. EDCs and Development of Endometriosis and Adenomyosis. Front Physiol 2022;12:807685. 10.3389/fphys.2021.807685 (Ref. 68); with permission.

#### 2.3.2 Inflammation, pain, and infertility overview

**FIGURE 4:**
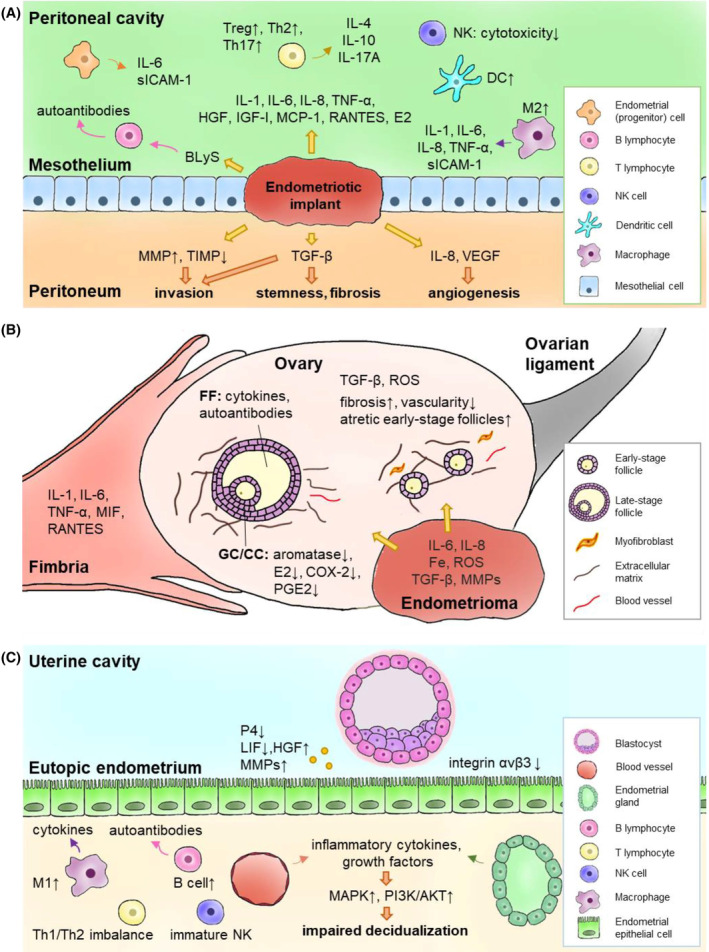
Models of inflammation leading to pain and infertility. Panel A: Inflammatory environment in the peritoneal cavity leading to disease formation and pain symptoms; Panel B: inflammatory environment affecting the ovary relevant to fertility; Panel C: inflammation in the uterine cavity leading to compromised embryo implantation and predisposing to poor pregnancy outcomes and dysregulated tissue homeostasis (See text for details). From Ref. [71]: Lin YH, Chen YH, Chang HY, Au HK, Tzeng CR, Huang YH. Chronic Niche Inflammation in Endometriosis‐Associated Infertility: Current Understanding and Future Therapeutic Strategies. Int J Mol Sci. 2018;19(8). 10.3390/ijms19082385, with permission.

Endometriosis causes intense inflammation in disease‐bearing compartments as well as systemically (Figure 4).^5^, ^56^, ^71^, ^72^, ^73^, ^74^, ^75^ Peritoneal fluid and endometriosis lesions contain multiple cell types and comprise a complex and dynamic environment dominated by inflammatory, angiogenic, and endocrine mediators (Figure 4A).^71^, ^76^, ^77^, ^78^ These mediators stimulate nociceptors and promote fibrosis and scarring, resulting in pelvic pain, a hallmark of the disorder.^79^ There are also alterations in peripheral and central nervous system pain processing, including visceral and central sensitization^79^, ^80^ that challenge current treatment strategies (see below). Anatomic distortion and adhesive disease in the pelvis, compounded by the inflammatory peritoneal milieu's adverse effects on oocyte quality, ovarian granulosa cell and sperm function, embryo development, and tubal motility (Figure 4B)^71^ are major contributors to subfertility associated with endometriosis.^71^, ^81^ Moreover, endometrium of endometriosis patients has a pro‐inflammatory phenotype of immune and mesenchymal cells with impaired progesterone signaling in the latter (Figure 4C),^59^, ^62^, ^63^, ^71^, ^82^, ^83^ believed to contribute to compromised embryo implantation and poor pregnancy outcomes in patients with disease.^61^ As inflammatory signals differ across age and various ethnic groups, expansion of these studies to diverse populations is warranted to achieve precision medicine.

#### 2.3.3 Single‐cell technologies

Endometrial and endometriosis bulk transcriptomics, epigenomics, proteomics, and metabolomic studies have been reported over the past decade, contributing to our understanding of mechanisms underlying the pathophysiology of the disease. These are summarized in several recent reviews.^7^, ^61^ Here, we focus on recent single‐cell transcriptomics and immunomics (CyTOF, imaging mass cytometry) of endometrium, endometriosis lesions, peritoneal fluid, and peripheral blood relevant to the pathophysiology of endometriosis.

##### 2.3.3.1 Single‐cell RNAseq

Single‐cell technologies are revolutionizing cell analyses across tissues and diseases, and an atlas of normal, cycling endometrium has recently been derived at single‐cell resolution by several groups.^84^, ^85^, ^86^, ^87^ These studies underscore heterogeneity of specific cell types and altered features in different hormonal states across the menstrual cycle and provide a key backdrop to studies on endometrium and endometriosis lesions at the single‐cell level. Recently, scRNAseq^88^, ^89^, ^90^, ^91^, ^92^ have complemented bulk tissue, blood, peritoneal fluid, and *in vitro* cellular and tissue organoid analyses of endometriosis lesions and endometrium from cases versus controls without disease (Table 2). These have provided further insights into the heterogeneity of cell types/subtypes, unique clusters, and signatures informing mechanisms and pathways involved in cellular dysfunctions relevant to pain and fertility compromise in patients with disease. Table 2 provides a summary of some recent single‐cell transcriptomic studies wherein samples were obtained in different hormonal milieus (menstrual cycle phase, exogenous hormones), and between 55 000 and 378 000 cells were sequenced. Other reports will likely follow suit soon.

**TABLE 2:** Recent single cell analyses of endometriosis lesions.

Reference	Tissue analyzed	Total cells sequenced	Technology/Platform	Total subjects	Endometriosis type and ASRM Stage	Controls	Race/Ethnicity	Hormones/IUD	Cycle phase endometrial histology	Main findings
Ma 2021	Endometrium, ovarian endometrioma	55 000	scRNAseq (10X)	*n* = 6 subjects (*n* = 3 cases, *n* = 3 controls)	ASRM Stage III, IV ovarian endometriomas	Healthy controls without endometriosis	N/A	None	All in proliferative phase	*Fibroblasts*: heterogeneous populations with clusters: cytokine. inflammatory response; FGF, immune response; ECM, cell adhesion; angiogenesis, hypoxia. All MAPK, TNF, IL‐17. TGFβ signaling, high expression of *StAR*. *Immune populations*: uNK cell frequency in EuE normal > EuE disease > endometrioma. Fewer T cells and uNK cells are more active, Mϕ enriched in tissue remodeling in lesion vs Eu E. *Conclusion*: FB and immune sub‐populations contribute a pro‐inflammatory, angiogenic environment in endometriomas.
Garcia‐Alonso 2022	Endometrium (functionalis, full thickness), endometriosis peritoneal lesions (red, white, black)	98 569	scRNAseq, snRNA seq, spatial profiling. Lesion microarray data (GSE141549)	*n* = 3 functionalis *n* = 6 full thickness; Microarray data controls: endom *n* = 42, peritoneum *n* = 12; *n* = 9 red, 9 white, 11 black	Peritoneal disease: red, white, black	Healthy controls (functionalis ayer) *n* = 6 full thickness without reproductive disorders; normal peritoneum	N/A	None	Proliferative, secretory; Microarray samples: Control Endo 17PE, 25SE; Perit 4PE, 8SE; Lesions: Red 2PE, 7 SE; White 5PE, 4SE; Black 6PE, 5 SE	Peritoneal lesions upregulated markers of PE (SOX9+ and pre‐ciliated) versus peritoneum and Upregulated markers for SOX9+LGR5+ subset (WNT7A, KRT17) as in PE. In contrast, secretory cell PAEP and SCGB2A2 and ciliated cell PIFO, TP73 epithelial markers, are ~ to peritoneum. *Conclusion*: Dysfunctional epithelium is a major driver of endometrial disease with two SOX9 populations dominant in endometriosis.
Shih 2022	Menstrual endometrium	43 054	scRNAseq (10X)	*n* = 33 subjects (*n* = 11 Dx, *n* = 13 sx, *n* = 9 controls)	N/A; another group with Sx but no Dx.	No endometriosis diagnosis. ? Other GYN disorders	White: 10 Dx, 13 sx, 7 controls Black: 0 Dx, 0 sx, 1 control Hispanic: 0 Dx, 0 sx, 0 cont Mixed: 0 Dx, 0 sx, 1 control Other: 1 Dx, 0 sx, 0 control	None on hormones, except 1 case used vaginal P4. Re‐analysis showed no impact on results	Menstrual (heaviest flow, mostly CD 1 or 2)	In cases, menstrual endometrial stromal cells displayed decreased decidualization markers. Menstrual endometrium displayed a marked reduction of uNK cells and enrichment of B cells. Subjects with symptoms but no diagnosis were similar to controls. *Conclusion*: menstrual endometrium reflects SE abnormalities in endometriosis and could be used for biomarker development.
Tan 2022	Endometrium, endometriosis lesions	122 000	scRNAseq (10X) IMC organoids	*n* = 27 subjects (*n* = 19 cases, *n* = 8 controls; *n* = 14 sequenced	ASRM Stages II‐IV, peritoneal lesions, ovarian "lesions", organoids	No endometriosis or inflammatory conditions	White: 9 cases, 3 controls; Asian: 4 cases, 2 controls; Hisp: 5 cases, 3 controls; Black: 1 case, 0 controls	Progestin [NETA, LVN, drospirenone, norelgestromin] ± ethinyl E2, provera, levonorgestrel IUD, copper IUD	wkly PE: 3 cases, 2 control; inactive 3 cases, 0 control; mens: 1 case, 0 control, exog hormone effect: 7 cases, 7 control PE: 0 cases, 2 controls IE: 0 cases, 1 control ESE: 4 cases, 0 control N/A: 1 case, 1 control	*Peritoneal lesions*: similar cell composition as EuE; dysregulated innate immune and vascular systems; Immune tolerant peritoneal niche involving Mϕ, DCs; unique perivascular mural cell type with angiogenic and immune cell trafficking properties; novel epithelial progenitor. *Ovarian lesions*: distinct cell compositions, transcriptomes. *Conclusion*: immune and vascular components of peritoneal endometriosis favor neo‐angiogenesis and an immune tolerant niche in the peritoneal cavity.
Fonseca 2023	Endometrium, endometriosis lesions, unaffected ovary, and peritoneum	373 851	digital scRNAseq	*n* = 21 subjects *n* = 17 cases, *n* = 4 controls *n*‐54 specimens collected	Cases: *n* = 17 Endometrium Endometrioma Superficial and deep disease *n* = 9 w/o GYN disorders; *n* = 8 w/ adenomyosis, uterine fibroids ± polyp	Controls *n* = 4 *n* = 3 PMP, 1 peri‐ menopause, all no evidence of endometriosis, all w/ leiomyoma ± adenomyosis ± uterine polyp	White: 13 cases, 2 controls Black: 2 cases, 0 controls Asian: 1 case, 0 control Other: 1 case, 0 control	*Cases*: *n* = 14 on no hormones. *n* = 1 w/ vaginal ring E+P; *n* = 1 on E/T, P4; *n* = 1 on E2+P4. *Controls*: 3 of 4 on E ± P4; NETA; *n* = 1 peri‐menopause in luteal phase	*Cases*: Proliferative phase: *n* = 7; secretory phase: *n* = 9; N/A (on hormones) *n* = 3. *Controls*: Proliferative *n* = 0, Secretory *n* = 1, N/A (on hormones) *n* = 3	Cell/molecular signatures of endometrial‐type epithelium and stroma differed across tissues c/w restructuring/transcriptional reprogramming in lesions. Eu E enriched in eEC, endothelium; endometriomas: enriched in B cells and plasma cells, lesions: in mast cells, T/NK‐T cells. Endometriomas: immune and C activation, some cells found only in pts on hormones. ARID1A mutation: pro‐(lymph)angiogenic, stroma/adjacent mesothelium pro‐inflammatory. Lesion ciliated epithelial signatures c/w ovarian cancer. Some histology‐negative mesothelial cells had disease signatures. *Conclusion*: scRNAseq gives insight into endometriosis phenotypes and depends on hormone status and could identify occult disease.
Huang 2023	Endometrium	128 243	scRNAseq (10X)	*n* = 10 sequenced *n* = 6 cases, *n* = 4 controls	ASRM Stages I, II	No endometriosis, benign ovarian cysts	N/A	None	EPE: 3 cases, 3 controls MSE: 3 cases, 3 controls LSE: 1 control	In MSE (WOI) of Stage I/II subjects, one epithelial cell cluster expressing PAEP and CXL14 was absent vs controls; Immune cells in controls decreased in SE, but no cycle variation of total, uNK, and T cells in cases was observed. Pro‐inflammatory cytokine expression was higher in endometrial immune cells in cases. *Conclusion*: Stage I/II disease is associated with lower epithelial receptivity markers, abnormal immune cell frequencies, and pro‐inflammatory WOI environment.

Abbreviations: ASRM, American Society for Reproductive Medicine; C, complement; CD, cycle day; c/w, consistent with; DC, dendritic cells; Dx, diagnosis; E_2_, estradiol; E, estrogen; eEC, endometrial epithelial; exog, exogenous; ECM, extracellular matrix; ESE, early secretory endometrium phase; EuE, eutopic endometrium; FB, fibroblast; FGF, fibroblast growth factor; IE, interval endometrium; IL, interleukin; IUD, intrauterine device; LSE, late secretory phase endometrium; LVN, levonorgestrel; mens, menstrual; Mϕ, macrophages; MAPK, mitogen activated protein kinase; MSE, mid‐secretory endometrium phase; N/A, not available; NETA, norethindrone acetate; P_4_, progesterone; PAEP, progesterone‐associated endometrial protein; PE, proliferative endometrium phase; sc, single cell; sn, single nuclei; sx, symptoms; RNA, ribonucleic acid; SE, secretory endometrium phase; seq, sequencing; StAR, steroidogenic acute regulatory protein; T, testosterone; TNF, tumor necrosis factor; uNK, uterine natural killer.

Studies restricted to a single menstrual cycle phase are highly informative about disease cell type signatures and phenotypes. For example, in the proliferative cycle phase, fibroblasts and immune cell subpopulations contribute to a pro‐inflammatory, angiogenic environment in endometriomas, and T cell and uNK cell frequencies are lower, uNK cells are more active, and macrophages (Mφ) are enriched and have features of tissue remodeling versus eutopic endometrium.^88^ García‐Alonso et al analyzed endometrium and full‐thickness tissue (functionalis and basalis) and leveraged bulk microarray transcriptomic data from endometriosis peritoneal lesions to characterize lesion cell types, compared with normal endometrium and peritoneum across the cycle (Table 2).^84^ They found upregulated markers in peritoneal lesions of SOX9+ and pre‐ciliated epithelial cells, a SOX9+/ LGR5+ subset as in proliferative endometrium, and similar expression of secretory cell PAEP and SCGB2A2 and ciliated cell PIFO and TP73 as in peritoneum. Dysfunctional epithelium as a major driver of endometrial disease with two SOX9 populations dominant in endometriosis is a foundational observation about disease pathogenesis and pathophysiology. Menstrual endometrium scRNAseq in endometriosis patients reveals decreased decidualization markers in stromal fibroblasts, reduced frequencies of uNK cells, and enrichment of B cells, demonstrating that menstrual endometrium reflects secretory endometrium abnormalities as possible biomarkers of disease.^89^ Another study on mid‐secretory endometrium (i.e., the window of implantation, (WOI)) of stage I/II subjects revealed that in one epithelial cell cluster PAEP and CXCL14 expression was absent, immune cells had higher pro‐inflammatory cytokine expression, and no cycle variation of uNK and T cell frequencies was observed versus controls.^91^ Lower epithelial receptivity markers, abnormal immune cell frequencies, and a pro‐inflammatory WOI likely adversely affect implantation and pregnancy outcomes in patients with stage I/II disease.

In a study of cases and controls on progestins, peritoneal lesions had similar cell compositions as eutopic endometrium but dysregulated innate immune and vascular components, in contrast to endometriomas with distinct cell compositions.^90^ Peritoneal disease displayed an immune tolerant niche involving Mφ and dendritic cells (DCs), a unique perivascular mural cell type with angiogenic and immune cell trafficking properties, and a novel epithelial progenitor. Overall, these data demonstrate that immune and vascular components of peritoneal endometriosis favor neo‐angiogenesis and an immune‐tolerant niche in the peritoneal cavity. ScRNAseq of endometrium and all three endometriosis lesion types versus control endometrium in patients treated with steroid hormones revealed different cell/molecular signatures of epithelium and stroma across tissues, consistent with restructuring/transcriptional reprogramming in lesions.^92^ Interestingly, endometriomas displayed immune cell and complement activation and were enriched in B cells and plasma cells, suggesting infection in endometriomas and a unique role for B cells which have received limited attention in endometriosis pathophysiology.^59^, ^93^, ^94^ Peritoneal disease was enriched in mast cells and T/NK‐T cells, and some histologically negative mesothelium surprisingly had disease signatures. ARID1A mutation in epithelia displayed pro‐(lymph)angiogenic, stroma/adjacent mesothelium pro‐inflammatory features and ciliated epithelial signatures consistent with ovarian oncogenic potential.

Overall, these studies provide important insights into features of endometrial and endometriosis cell types at single‐cell resolution, their heterogeneity, cell‐cell interactions, and in some cases, spatial localization. Moreover, unique targets, pathways, and signatures can be mined for future diagnostic and novel therapeutic development. These studies also underscore challenges in conducting studies on endometriosis tissue from patients and controls. These include the importance of well‐defined clinical metadata (cycle phase/hormonal status, medications, comorbidities); standard operating procedures in tissue processing^95^, ^96^, ^97^, ^98^, ^99^; defining lesion types and co‐existing lesion types at sampling that could affect results; high prevalence of co‐existing common gynecologic disorders (uterine fibroids, adenomyosis) in both cases and controls; defining the control group (no endometriosis and without or without other gynecologic disorders); mixing natural cycles with various hormonal treatments, and recognizing that ovarian‐derived and synthetic hormones (e.g., progesterone and progestins) while signaling through common pathways also signal via unique pathways that could influence outcomes.^100^, ^101^ While numbers of cells sequenced enrich the phenotypic features of individual cell types, the numbers of subjects recruited is, by comparison, low, and notable is the limited diversity of the cohorts recruited (Table 2). To date, as most studies either did not describe ethnicity or had a preponderance of White subjects, the data across ethnicities are limited and offer opportunity to close the gap in future research.

##### 2.3.3.2 IMC and CyTOF

Mass cytometry (cytometry time of flight (CyTOF), a multi‐parameter single cell technique, has recently been applied to characterize and quantify immune cell populations in peritoneal fluid,^102^ peripheral blood,^102^, ^103^ and eutopic endometrium^103^ of patients with versus without endometriosis. More than 40 distinct immune cell types were found in peritoneal fluid and stratification by disease stage collectively underscore a complex, dynamic, and heterogeneous, inflammatory microenvironment in the pelvic cavity of patients with endometriosis.^102^ CyTOF also revealed enrichment and activation of distinct populations in different menstrual cycle phases and endometriosis disease stages and controls and demonstrated dysregulation, in particular, of the mononuclear phagocytic system in endometrium and peripheral blood in patients with endometriosis and offering candidates for diagnostic and therapeutic target development.^60^

### 2.4 Diagnosis

#### 2.4.1 Surgery and staging

Surgery has been the gold standard to diagnose endometriosis with visualization and histologic confirmation of endometrial glands, stroma, and/or hemosiderin‐laden macrophages in suspected lesions biopsied at laparoscopic or robotic surgery.^4^ About 67% of suspected lesions are confirmed histologically,^104^ depending on appearance, size, and disease stage, with variability among surgeons in identifying uncommon lesion types.^105^ Computer‐aided histopathologic characterization of endometriosis lesions is transforming this landscape. A recent classifier using digitized tissue slides and quantification of stromal and epithelial markers found different cell ratios in deep versus superficial disease and versus endometriomas and significant correlations with pain (*p* < .0005).^106^ Recently, molecular imaging and spatial characterization of endometriosis tissues using desorption electrospray ionization mass spectrometry (DESI‐MS) and statistical modeling allowed classification of disease lesions with overall accuracies of 89.4%, 98.4%, and 98.8% on training, validation, and test samples.^107^ Incorporating histologic software and advanced imaging techniques into standard diagnostic pipelines may improve endometriosis diagnosis and provide prognostic and personalized therapeutic options.^106^

The revised American Society for Reproductive Medicine (rASRM) scoring system, constituted at surgery, quantifies disease burden (except deeply invasive disease) and accompanying pelvic adhesions.^108^ The rASRM stages are numerical tallies of disease burden and adhesion scores and range from stage I (lowest) to stage IV (highest) scoring, although scores do not correlate with pain scores or responses to medical therapies for pain or infertility. The AAGL 2021 Endometriosis Classification allows for identification of objective intraoperative findings to discriminate surgical complexity but similarly does not correlate with pain scores, responses to medical therapies, or fertility.^109^ The World Endometriosis Society (WES) 2017 consensus statement^110^ recommends using a “classification toolbox” that includes the rASRM system and the Enzian system for deep disease to improve disease classification. It also promotes using the extensively validated Endometriosis Fertility Index (EFI), which has high sensitivity and specificity for fertility outcomes *after* surgical treatment^111^ and greatly facilitates fertility therapy planning. Surgery as a method of diagnosis has its limitations overall and access to laparoscopic or robotic surgery is not equal.^112^, ^113^ Please see section under Disparities for more details.

#### 2.4.2 Shifting away from surgical diagnosis

As surgical diagnosis has contributed to the 7‐ to 11‐year latency between first symptom onset and surgical treatment of symptomatic patients with endometriosis,^114^ the diagnostic paradigm is now shifting to a multi‐modal approach.^115^ This includes integrating extensive medical, menstrual, pregnancy, surgical, family, medication, lifestyle, and environmental histories, physical examination, and imaging prior to initiating medical therapies for pain and/or infertility. Several professional organizations across the globe endorse this approach, including the American College of Obstetricians and Gynecologists (ACOG), WES, the Society for Obstetrics and Gynecology of Canada, and the European Society for Human Reproduction Embryology (ESHRE).^116^, ^117^

#### 2.4.3 Gynecologic history

**FIGURE 5:**
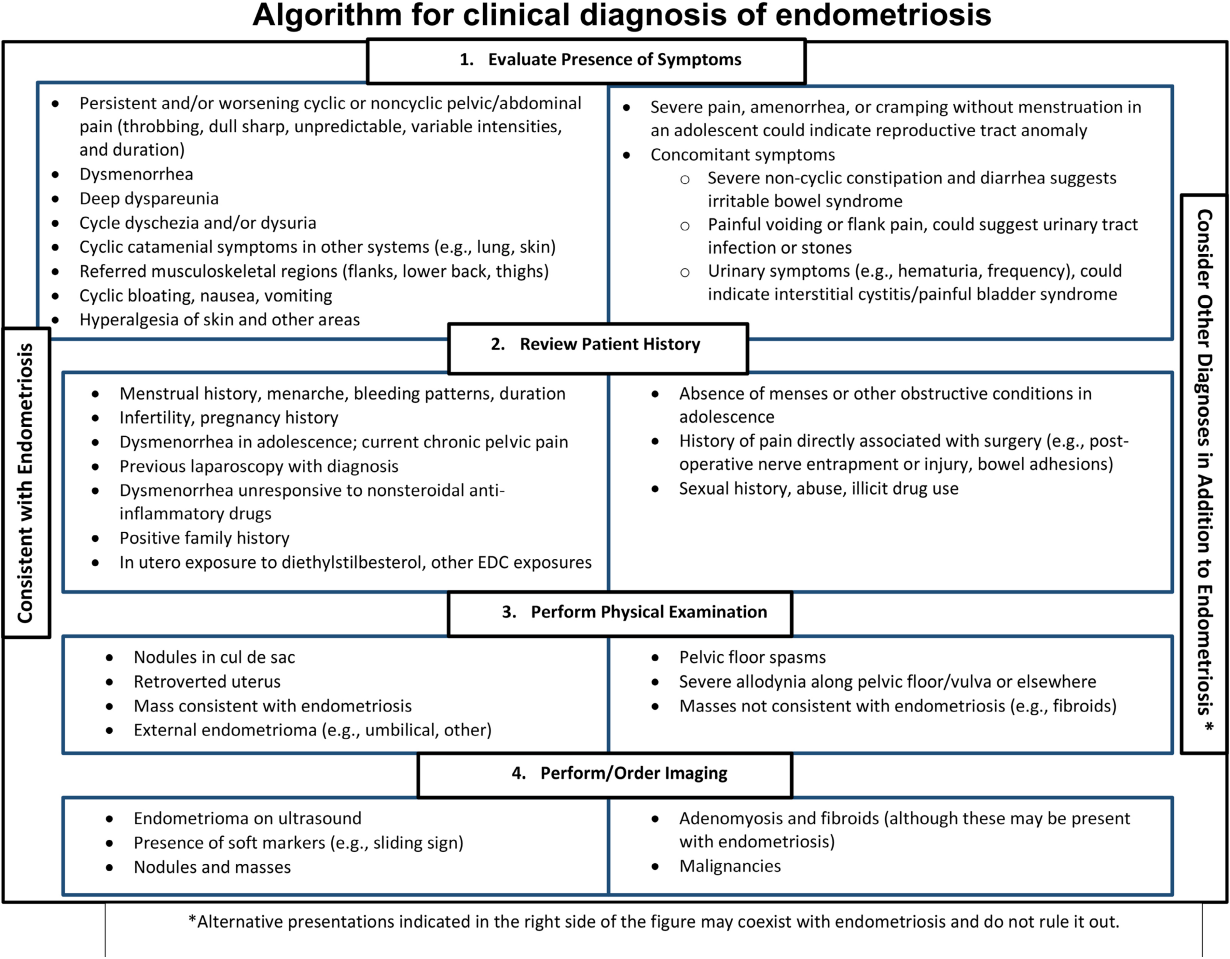
Algorithm for clinical diagnosis of endometriosis. The algorithm shows sequence of clinical diagnosis beginning with evaluation of symptoms (section 1), review patient history (section 2), perform physical examination (section 3), and performing/order imaging (section 4). Items on the left side of the figure are informative about possible endometriosis diagnosis. Items on the right side may co‐exist with endometriosis but do not rule it out. From Ref. [115]: Agarwal SK, Chapron C, Giudice LC, et al. Clinical diagnosis of endometriosis: a call to action. Am J Obstet Gynecol. 2019;220(4):354.e1‐354.

The most common pain pattern in patients with endometriosis is dysmenorrhea (pain with menses) beginning at menarche, disrupting school and other activities, and worsening over time to unpredictable non‐menstrual pelvic pain.^7^ However, these symptoms, along with other common symptoms, e.g., gastrointestinal dysfunction and bladder pain, overlap with other disorders. Figure 5 shows an algorithm for clinical diagnosis of endometriosis that can distinguish endometriosis from other conditions.^115^ Patient and family history, symptoms, and findings on physical examination increase the likelihood of an endometriosis diagnosis. For example, the odds ratio for endometriosis diagnosis, based on only pain symptoms, increases from 5.0 to 84.7 for 1 and >7 symptoms, respectively.^118^ Pre‐menstrual spotting, irregular periods, and occasional heavy menstrual bleeding are relatively infrequent presentations.^115^ Nonetheless, these are all important components of history‐taking in symptomatic patients and can validate the known varied symptoms experienced by those with disease and prompt investigation into other disorders.^119^ However, there is a lack of awareness about endometriosis among patients and providers influenced by societal and cultural normalization of pain in women and stigma surrounding menstrual cycles.^120^ This may lead to underreporting, especially among underrepresented racial and ethnic minorities and those of lower socioeconomic status.^121^, ^122^

#### 2.4.4 Pain instruments

The visual analog scale (VAS) is a validated acute and chronic pain instrument with paper‐, laptop computer‐, and mobile phone‐based platforms that can facilitate data collection and entry into the electronic medical record symptom course over time. The International Pelvic Pain Society's Pelvic Pain Assessment form is a comprehensive clinical assessment of patient symptoms replete with pain maps and extensive gynecologic and health history intake (http://www.pelvicpain.org). Although it is not designed to diagnose endometriosis specifically, it is helpful for clinicians to visually understand distribution of pain and patterns, and it can be empowering for patients to document their pain history, location, and quality. This could be achieved with the help of mHealth—the use of mobile devices for health care. Since 2013, there have been at least 26 mHealth applications (“apps”) focused on endometriosis and chronic pelvic pain that feature various functionalities and are available through the Apple iTunes Store, Google Play, and/or BlackBerry World. Among these mHealth apps, 16 (61.5%) serve as educational tools, focusing on symptoms and how to avoid or deal with them and/or improve general quality of life. Nine of the apps (34.6%) provide information about endometriosis diagnosis, management, and treatment. Seven apps (27%) are for social networking and allow users to share their stories and experiences. Eight apps (31%) function as a diary, allowing users to record menstrual cycle and symptom information. As with many mHealth apps, there are appreciable concerns pertaining to the lack of evidence‐based medicine and/or medical professional involvement that need to be addressed by these mHealth apps for endometriosis and chronic pelvic pain; nevertheless, these apps have the potential to serve as a valuable resource for patients and clinicians.^123^ For example, Phendo is a research mHealth app designed for Apple and Android mobile devices for users to self‐track their experiences of endometriosis, including questions related to pain, menstruation, bleeding, gastrointestinal (GI) and genitourinary symptoms, and other symptoms.^124^ Analysis of user data collected by the Phendo mHealth app has found that those who habitually exercise at least ~three times per week are less likely to report pain symptoms after having exercised on the previous day.^125^ The Phendo app can assist not only with informing recommendations for self‐management of pain from endometriosis but also can contribute to better phenotyping and understanding of this disease.^124^

#### 2.4.5 Pelvic examination

Pelvic examination may identify endometriosis with high accuracy, although it is highly dependent on disease location, does not detect superficial peritoneal lesions, and may be unacceptable in non‐sexually active patients.^115^ Ruling out other causes of pelvic pain and infertility is essential, as therapies for these likely differ and may require referral for specialty care.

#### 2.4.6 Infertility

As 30%–50% of persons with a uterus and infertility have endometriosis, and as treating the disease can affect fertility outcomes, diagnosing endometriosis is an important part of the infertility workup in addition to patient age, duration of infertility, prior pregnancies and their outcomes, comorbidities, and male partner evaluation.^126^ Women with ovarian endometriomas may have decreased ovarian reserve (see below), and prompt diagnosis is key in planning fertility therapies and/or surgical extirpation, if warranted. Thus, a high index of suspicion is warranted for endometriosis in infertility evaluation and care.

#### 2.4.7 Imaging

Imaging technologies are commonly used to evaluate the pelvis for endometriosis as well as other gynecologic disorders that can mimic endometriosis symptoms, e.g., ovarian cysts and uterine fibroids, and are increasingly being used to diagnose endometriosis, adjunctively with patient symptoms and history.^127^ The first‐line imaging approach is transvaginal ultrasound (TVUS),^128^ which has high sensitivity and specificity for ovarian endometriomas and can reveal immobility of pelvic structures due to adhesions and fibrosis. However, it performs poorly in detecting superficial peritoneal disease. TVUS has high accuracy, comparable to MRI, to detect deep infiltrating disease with experienced sonographers and enhancing protocols and is valuable in pre‐operative assessment and surgical referral.^127^ A Cochrane meta‐analysis found that TVUS has sensitivity and specificity similar to surgical diagnosis, depending on the type of disease.^116^ A model combining patient history, symptoms, and ultrasound predicts rASRM stages III/IV but has not been widely adopted as it has low accuracy in predicting stage I/II disease.^114^ A systematic review and meta‐analysis of 30 studies involving 4,565 patients reported comparable accuracy for TVUS, trans‐rectal (TR)US, and MRI to diagnose endometriosis, and greater than physical examination alone (Table 3).^129^ It is anticipated that as more data accrue and are validated, advanced computational analyses and further predictive modeling will result in algorithms to diagnose endometriosis with high accuracy. Of course, these approaches would need to be established and validated across diverse patient cohorts. This would be helpful to clinicians and to researchers, especially as surgical diagnosis currently is required by the U.S. Food and Drug Administration (FDA) for clinical trials assessing safety and efficacy of medications for endometriosis‐related dysmenorrhea and non‐menstrual pelvic pain.

**TABLE 3:** Performance of various imaging modalities and physical exam in the diagnosis of endometriosis.

Condition	Modality	Sensitivity (%)	Specificity	Likelihood ratio	Accuracy	Author^a^
Deep endometriosis	TVUS	78.5	95.2			Bazot 2004
Deep bowel	TVUS	91	98	+LR: 30.36‐LR: 0.09		Hudelist 2013^b^
Recto‐sigmoid	TVUS	98	100		100%	Abrao 2007
	MRI	83	98		90%	
Intestinal endometriosis	TVUS	86	73			Saba 2012
	MRI	90	73			
Deep endometriosis	TVUS	76	94		AUC 0.92	Zhang 2020^b^
	MRI	92	87		AUC 0.91	
	TRUS	91	80		AUC 0.93	
	PE	71	69		AUC 0.76	

Abbreviations: AUC, area under the curve; LR, likelihood ratio; MRI, magnetic resonance imaging; PE, physical exam; TRUS, transrectal ultrasound; TVUS, transvaginal ultrasound. Adapted from Zhang et al 2020 (Ref. ^129^), with permission.

^a^ Author citation in Zhang et al 2020 (Ref. ^129^).

^b^ Pooled performance percentages from systematic reviews/meta‐analyses.

### 2.5 Molecular biomarkers of disease

Despite extensive research over the past two decades, disease‐specific biomarkers have yet to be identified and validated in multi‐site clinical trials to diagnose and/or stage endometriosis and meet or exceed the sensitivity (94%) and specificity (79%) of the gold standard, laparoscopy.^130^ That said, advances are being made to identify biomarkers in eutopic endometrium, blood (plasma, serum, menstrual), and saliva. Table 4 summarizes current biomarkers under evaluation, most of which have relevance to disease pathophysiology and some of which may be more acceptable to patients (e.g., blood test versus endometrial sampling). Disease biomarkers are anticipated to shorten the time to diagnosis and thus early clinical intervention, follow disease progression and recurrence, and assess response to treatments. Moreover, biomarkers will provide affected persons with a timelier diagnosis and empowerment to seek immediate support and multidisciplinary care for their symptom management and well‐being. A non‐surgical method of diagnosis may also help to bridge the gap in access to laparoscopy for marginalized communities.

**TABLE 4:** Diagnostics being developed.

Source/Company	TM test name	Sampling	Technology	Status	NCT #
NIH Protocol	EndoMarker Protocol	Plasma, serum EBx all cycle phases	ELISA cytokines, RNA expression	Complete *n* = 114 2020 Clin Trail 2019 publication	31061704
CiceroDx	Receptiva	EBx timed to LH surge	BCL6	WOI inflammation	N/A
			IHC		
			H‐score		
NextGen Jane		Menstrual blood (tampon)	RNA, miRNA	SBIR funding	
				*n* = 189 clinical trial/validation	
Aspira	EndoCheck	Venous blood	Protein biomarkers neural network	*n* = 600 case control vs. laparoscope launch 2023	52455695
DotLab	DotEndo	Venous blood (saliva)	miRNAs	Completing multicenter EMPOWER *n* = 750	4598698
				2024	
Ziwig	ENDOTEST	Saliva	miRNAs	*n* = 1000 multicenter trial	5244668
				Late 2022	
				Early 2023	
Scailyte AG	ScaiVision‐Endo	Venous blood (PBMCs), Ebx	Single cell technologies	*N* = 100; initiate trial	N/A
				Late 2022	
				Early 2023	
Hera Biotech	MetrixDx	EBx	Connexin proprietary	*N* = 75	5698212
				Study start 2022, anticipated end 2023	
Endometrics	EndoCup	Menstrual blood (cup)	Published on RNA expression; technology proprietary	In process	None
Endogene Bio		Menstrual blood	Epigenetic markers	Under construction	N/A

Abbreviations: BCL6, B cell leukemia 6; EBx, endometrial biopsy; ELISA, enzyme‐linked immunoassay; IHC, immunohistochemistry; LH, luteinizing hormone; mi, micro; NCT#, US National Institutes of Health Clinical Trial number; PBMCs, peripheral blood mononuclear cells; RNA, ribonucleic acid; TM, trademark; WOI, window of implantation.

#### 2.5.1 Endometrial biomarkers

As endometrium is the origin of pelvic endometriosis and has cellular features and molecular pathways that differ in patients with and without disease at the transcriptional and epigenetic levels,^3^ it has been mined for possible disease diagnosis and staging classifiers—e.g., the EndoMarker^TM^ protocol for sampling endometrium and concomitant blood, all cycle phases^131^ and specific machine learning classifiers for transcriptomics and methylomics.^132^ Endometrial gene expression (oligonucleotide microarrays, bulk RNA‐sequencing, scRNAseq, Q‐RT‐PCR),^89^, ^133^, ^134^, ^135^ and endometrial whole DNA methylome and candidate gene DNA methylation signatures^136^, ^137^, ^138^, ^139^, ^140^ have identified genes involved with steroid hormone dependence and abnormalities in patients with versus without endometriosis. However, most results fail to be replicated due to limited sample size, cellular heterogeneity in bulk tissue, poor cycle phase assignments, and limited clinical metadata. As menstrual cycle phase is a main driver of endometrial gene expression, diagnostic development at a specific cycle phase or phase‐independent classifiers would be preferred. The EndometDB with expression data from 115 patients and 53 controls and over 240 000 genes and clinical features is a valuable resource.^141^

#### 2.5.2 Window of implantation

It is known that endometrium in patients with endometriosis has a pro‐inflammatory environment manifested by heightened ERβ signaling and progesterone (P_4_) resistance, resulting in abnormal expression of several genes and proteins in the window of implantation, cycle days 20–24.^62^, ^63^ B‐cell lymphoma 6 (BCL6) was recently found to be abnormally upregulated in endometrium of patients with disease (and unexplained infertility some later identified with endometriosis).^142^ In patients with abnormal BCL6 protein expression, suppression of disease and associated inflammation using GnRH analogs or progestins, or surgical ablation reportedly improved live birth rates (50% treated, 7% untreated) after assisted reproduction (in vitro fertilization‐embryo transfer (IVF‐ET).^143^ Utility of endometrial BCL6 expression as a diagnostic tool in general and in terms of infertility related to inflammatory disorders (e.g., hydrosalpinx, adenomyosis)^144^ remains to be determined in clinical trials. The BCL6 endometrial test (“Receptiva”), based on H‐score immunohistochemical evaluation and timed to the LH surge, is currently marketed by CiceroDx^TM^ (https://receptivadx.com/).

#### 2.5.3 Menstrual endometrium

Endometrial biopsy is less invasive than laparoscopy for diagnosis, and recently menstrual blood has been a focus for diagnostic development. It contains shed endometrial cells largely reflecting molecular characteristics of secretory endometrium.^89^, ^145^ Currently, menstrual blood collected in a Smart Tampon^TM^ and assessed for RNA and miRNA expression is being developed by NextGen Jane (https://www.nextgenjane.com/), and menstrual blood collected in a menstrual cup by Endometrics (https://endometrics.us/) is also being developed for non‐invasive diagnosis of endometriosis.

#### 2.5.4 Circulating biomarkers

##### 2.5.4.1 Cancer Antigen 125 (CA125)

CA125 is expressed in endometrium and endometriosis lesions and is elevated in serum of some patients with disease.^146^ Serum CA125 >30 units/ml has overall specificity of 92.7% and sensitivity of 52.4% for all rASRM stages (I‐IV) and lower sensitivity for stage I/II versus III/IV disease (24.8% versus 63.1%, respectively).^146^ Thus it has minimal clinical value, although it is sometimes used to monitor changes in disease burden/recurrence in patients where imaging is not informative and surgery is contraindicated for medical or other reasons

##### 2.5.4.2 MicroRNAs

MicroRNAs (miRNAs) have been implicated in endometriosis pathophysiology,^147^, ^148^ and recently, classifiers were developed based on specific serum miRNAs to diagnose endometriosis in patients undergoing benign gynecologic surgery.^149^ These classifiers had high accuracy (AUC = 0.94), were validated in independent datasets, and distinguished rASRM stages I/II and III/IV from controls but not stages I/II from III/IV. Of those identified with disease, 90% had pelvic pain and 10% had infertility, and notably, diagnosis was independent of cycle phase or hormonal medications. Dot Labs (https://www.dotlab.com/) is developing plasma‐ and saliva‐based test for endometriosis through its “EMPOWER” study registered at NIH www.clinicaltrials.gov (NCT #4598698), with ongoing recruitment.

##### 2.5.4.3 Protein biomarkers

A study by Aspira (https://aspirawh.com/clinical‐studies/) is underway to develop EndoCheck^TM^, a test to diagnose endometriosis in which blood protein biomarkers are compared to laparoscopy, through NCT #5244668 with on‐going recruitment.

##### 2.5.4.4 Peripheral blood monocytes (PBMCs)

Scailyte AG (https://scailyte.com) is developing ScaiVision‐Endo^TM^, a venous blood study of PBMCs and endometrial biopsies, using single‐cell technologies, artificial intelligence, and multi‐omics analyses. Recruitment is ongoing.

##### 2.5.4.5 Circulating cell‐free DNA (ccf‐DNA)

Significantly elevated ccf‐DNA in patients with minimal/mild endometriosis versus controls with no disease was first reported in 2009, with discrimination between cases and controls and with receiver operating characteristics revealing 70% sensitivity and 87% specificity.^150^ Recently, however, endometrial and circulating cf‐DNA during menses was not found to differ between cases and controls.^151^ Protocols for ccf‐DNA isolation and quantification^152^ may account for the observed differences. Whether ccf‐DNA can be developed as a marker of endometriosis disease and perhaps stage remains to be determined.

##### 2.5.4.6 Salivary biomarkers

In addition to plasma miRNA studies, recently, a suite of 109 salivary miRNAs have been shown to diagnose endometriosis with high sensitivity, specificity, and AUC (96.7%, 100%, and 98.3%).^153^ ENDOTEST^TM^ is an miRNA‐based salivary test for endometriosis, currently under development by Ziwig (https://ziwig.com) through NCT #5244668.

These diagnostic candidates, based on endometriosis pathophysiology, show great promise and await multicenter randomized control trials for further validation and broader applications, e.g., assessing disease phenotypes and subtypes and disease and symptom recurrence across the lifespan. Testing of these biomarkers in diverse populations is warranted before they can be advanced to clinical practice. Furthermore, applications of computational predictive modeling approaches to these diverse types of molecular data can enable more precise diagnostic strategies.

### 2.6 Clinical treatments

**FIGURE 6:**
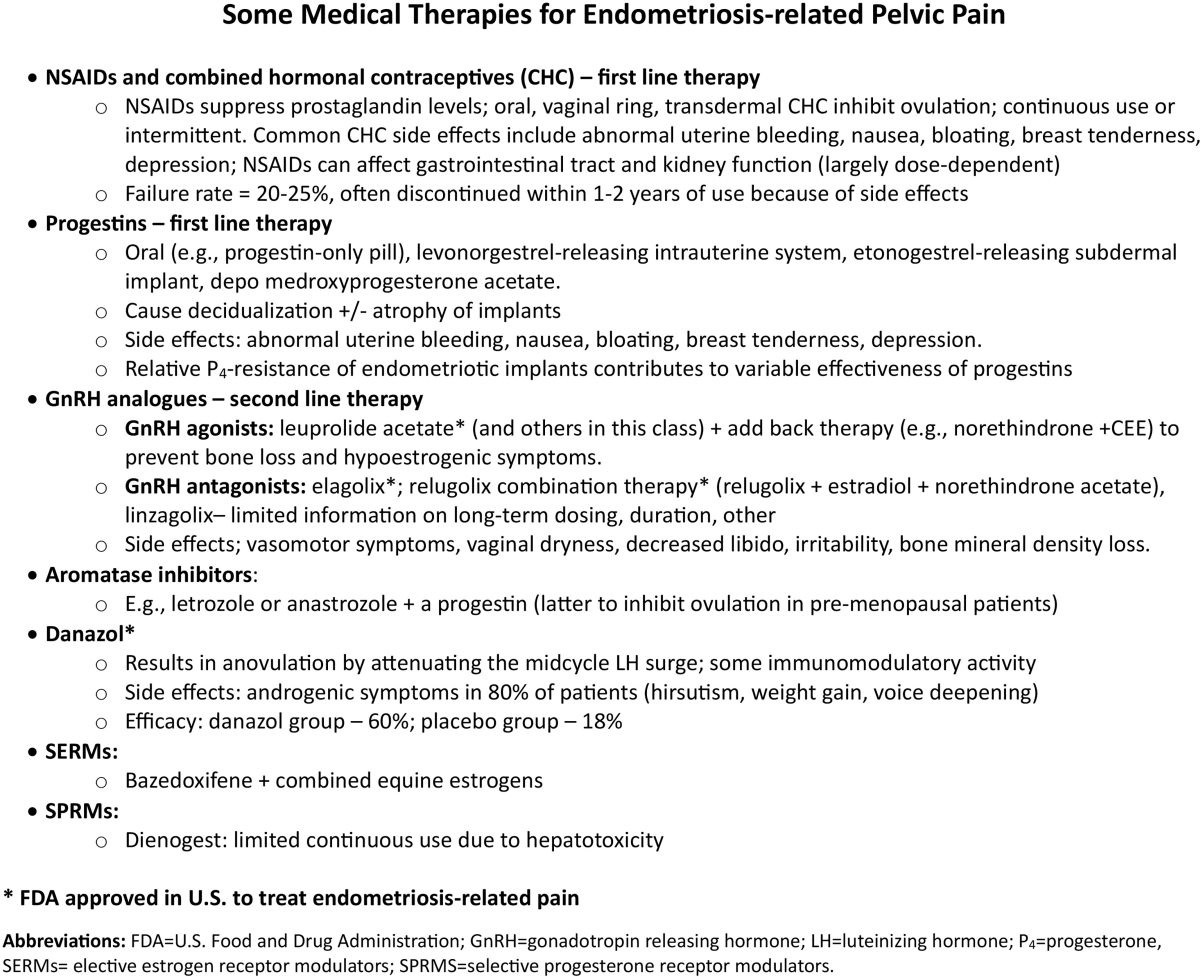
Some medical therapies for endometriosis‐related pelvic pain. The figure shows classes of drugs to treat endometriosis‐related pain, ranging from NSAIDs, CHCs, progestins, GnRH analogs, aromatase inhibitors, danazol, SERMs, and SPRMs. CHCs, combined hormonal contraceptives; GnRH, gonadotropin‐releasing hormone; LH, luteinizing hormone; NSAIDs, non‐steroidal anti‐inflammatory drugs; P_4_, progesterone; SERMs, selective estrogen receptor modulators; SPRMs, selective progesterone receptor modulators.

Leading professional groups have issued evidence‐based guidelines for managing symptoms of pain and infertility related to endometriosis,^117^, ^119^, ^154^, ^155^, ^156^ and there is high concordance among them. For pain management, medical therapy is usually the first approach (Figure 6), although surgery may be first‐line with or without post‐operative medical therapy, depending on the presentation and extent of symptoms.^117^

#### 2.6.1 Medical therapy for endometriosis‐related pelvic pain and infertility

##### 2.6.1.1 Pain

As endometriosis is estrogen‐dependent, therapies for associated pelvic pain mainly include opposing estradiol (E_2_) action or decreasing its circulating levels using contraceptive steroids, progestins, GnRH analogs, and aromatase inhibitors,^3^, ^7^ along with NSAIDs to minimize inflammation (Figure 6). While most of these approaches are initially effective in ~70% of patients with endometriosis‐associated chronic pelvic pain and dysmenorrhea, they lose effectiveness over time or are discontinued due to intolerable side effects.^3^, ^7^ Some have been variably reported to minimally reduce lesion size.^117^ Recently, new GnRH antagonists have shown 50–70% response rate for the co‐primary endpoints of dysmenorrhea and non‐menstrual chronic pelvic pain.^114^, ^157^, ^158^ These “new” oral drugs are variations on the theme of current hormonal treatments to date and exhibit unpredictable individual response and variable pain relief. In addition, evidence is limited regarding dosage and duration for long‐term use of the GnRH antagonists and the need for hormonal (estrogen and progestin) add‐back therapy to manage hypoestrogenic symptoms.^117^ Selective progesterone receptor modulators (SPRMs) have been used but have hepatotoxicity that limits continuous therapy, and selective estrogen receptor modulators (SERMs) have found applications in select cases coupled with GnRH agonists for severe and medially recalcitrant endometriosis‐related pain.^159^ Novel approaches are being pursued, including mining transcriptomic data and using a drug repositioning pipeline (see below).^160^

Kinase signaling pathways have been targets for endometriosis‐related pain, including, IKkb/NFkB, MAPK (ERK1/2, p38, JNK), and PI3K/AKT/ mTOR,^161^ although the current generation of kinase inhibitors carry potential for significant adverse side effects. Several immunomodulatory agents have also been evaluated for endometriosis‐related pain, infertility, and lesion size in either completed or ongoing clinical trials in patient and in animal models. These include anti‐TNFa agents (etanercept, infliximab), cytokine therapies (recombinant IL‐2, interferon‐a‐2b); angiogenesis inhibitors (simvastatin, quinagolide, cabergoline), immunomodulatory and anti‐inflammatory agents (pentoxifylline, pioglitazone, rosiglitazone, metformin, resveratrol, ECCg), and antioxidants (vitamin E, vitamin C, melatonin), with variable results.^71^ Recently long‐acting anti‐IL‐8 antibody therapy was shown to improve fibrosis and inflammation and decreased nodular lesion volume in an animal model of endometriosis.^162^

Having non‐hormonal medical therapies for endometriosis‐related pain, inflammation, and disease burden would be transformational and several candidates show great promise. However, the heterogeneity of the disease is a challenge to clinical development of many of these agents, as well as need to consider that most patients with endometriosis are of reproductive age, and thus agents that could affect fetal development would be challenging to assure safety in an undiagnosed pregnancy. Surgical treatment, sometimes combined with hormonal treatments, involves removal of endometriosis lesions, with recurrence in ~50% of women within 2–5 years.^163^ How surgical treatments would complement some of the candidates under development is a great opportunity for the future. However, to assure equity across populations, all studies need to recruit diverse subjects for analyses.

Thus, a major unmet need for symptom relief in patients with endometriosis is to develop novel drugs that target specifically disease‐associated pathways and abandon the historic and less than satisfactory “one size fits all” approach that continues today. As subtypes of disease lesions are diverse in their invasiveness, growth rates, pain attribution, and steroid hormone response, the need for precise treatments based on molecular basis of disease is well founded.

##### 2.6.1.2 Infertility

As endometriosis‐related infertility derives from anatomic distortion/adhesions and ovarian and endometrial dysfunction (see above), surgical approaches attempt to restore normal anatomy, while medical approaches attempt to minimize inflammation and improve the microenvironment in ovarian follicles and the endometrium for fertilization and embryo implantation, respectively. Medical therapies to manage pain mostly suppress the menstrual cycle and are contraindicated to treat endometriosis‐related infertility. Rather, medically assisted reproduction (MAR) (i.e., ovarian stimulation with anti‐estrogens (e.g., clomiphene) or aromatase inhibitors or injectable gonadotropins), accompanied by intercourse or intrauterine insemination are recommended as first‐line approaches.^117^ However, as female partner age is a key driver of fertility, depending on patient age, and with failed MAR approaches, in vitro fertilization and embryo transfer (IVF‐ET) and/or surgery are recommended, with shared decision‐making with the patient/couple.^117^ Expression of endometrial biomarkers in the window of implantation, such as BCL6, has led to treating patients with 2–3 months of gonadal suppression or surgery to remove endometriosis prior to IVF.^143^ While the results of enhanced pregnancy rates are promising,^143^ validation awaits outcomes of ongoing randomized controlled trials.

##### 2.6.1.3 Computational approaches to drug discovery

The molecular complexity and multifactorial nature of endometriosis pose unique challenges to the development of effective therapies and suggest the need for precision medicine that takes into account individual variability in genetic and other molecular measurements. This applies to medical therapies for endometriosis‐related pain as well as infertility. Since developing new drugs for a complex disease such as endometriosis takes a long time and involves huge costs, there is a pressing need to consider unconventional drug development strategies and precision medicine approaches, such as repositioning drugs currently used for other conditions. The approach of computational drug repositioning has a number of advantages over the development of new drugs and has been done successfully for various disease conditions. The development and availability of large‐scale genomic, transcriptomic, and other molecular profiling technologies in publicly available databases, in combination with the deployment of the network concept of drug targets and the power of phenotypic screening, provide an unprecedented opportunity to advance rational drug repositioning and data‐driven development of drug combinations based on the ability of single or multiple therapeutic agents to perturb entire molecular networks away from disease states in cell‐based and animal models. We and others have used aforementioned approaches to identify new uses for existing drugs for a number of different indications including inflammatory bowel disease,^164^, ^165^ cancer,^166^ Alzheimer's disease,^167^ COVID19,^168^ and most recently endometriosis.^160^ Genomic and transcriptomic technologies allow us to extract large amounts of data from patient samples, elucidating previously unknown factors involved in disease, which could lead to identifying new therapeutic strategies. As we learn more about the complex mechanisms associated with endometriosis and its related comorbid conditions, it is increasingly clear that treatments will likely require both precision medicine and combination therapeutic approaches.

#### 2.6.2 Surgical therapy for endometriosis‐related pelvic pain and infertility

##### 2.6.2.1 When to do surgery

Surgery remains a mainstay of current treatment for endometriosis.^163^, ^169^, ^170^, ^171^, ^172^, ^173^, ^174^, ^175^, ^176^, ^177^, ^178^, ^179^, ^180^, ^181^, ^182^, ^183^, ^184^, ^185^, ^186^, ^187^, ^188^ In general, surgery is indicated in symptomatic patients failing, unable to tolerate or declining medical regimens, in those attempting to conceive, and/or for infertility, and to exclude malignancy in the case of an adnexal mass.^117^ In general, laparoscopic or robotic surgery is favored even in the setting of advanced disease given associated shorter recovery and hospitalization, decreased cost, and safety compared to laparotomy.^169^ Surgery is as effective as medical therapy with many (73%) but not all of patients reporting symptomatic pain relief at 6 months compared to those undergoing diagnostic laparoscopy alone (21%).^170^ Furthermore, data on the effect of surgery on fertility outcomes are mixed.^169^

The goal of surgery is to remove all visible lesions of endometriosis and to restore normal anatomy.^163^, ^169^, ^170^ Surgical treatments for endometriosis vary among surgeons and include excision, fulguration, or laser ablation of endometriotic lesion on the peritoneum, excision or drainage of ovarian endometriomas, resection of deep infiltrating nodules, lysis of adhesions, and interruption of nerve pathways generally by traditional or robotic laparoscopy.^2^, ^163^, ^171^ In addition, hysterectomy and/or oophorectomy are often performed for those who have completed their family or do not desire fertility or uterine conservation. Surgeries for advanced stage or deep infiltrating endometriosis (DIE) can involve extensive adhesions, fibrosis, and invasion into important structures such as the bowel, bladder, diaphragm, or ureter requiring an advanced level of surgical training/expertise or a multidisciplinary team. Immediate complication risks include injury to the bowel, bladder, ureter, neurovascular bundles, and diaphragm and occur in 0.1% (3/1894) of major and 3.6% (3/84) of deeply infiltrative endometriosis surgeries.^172^ Complications can also affect long‐term quality of life and include fistula formation and bowel or bladder dysfunction due to iatrogenic denervation. Therefore, the decision of when to offer surgery is complex and not without careful consideration.

##### 2.6.2.2 Pain

Recent studies have attempted to answer this question for the indications of pain and fertility. A 2020 systematic review and meta‐analysis of 12 eligible studies addressing outcomes of endometriosis surgery (pain (*n* = 6), fertility (*n* = 7), quality of life (*n* = 1), and disease progression (*n* = 3)), and patient preference (*n* = 7) reported an improvement in overall pain at 6 months compared to diagnostic laparoscopy, risk ratio [RR: 2.65 (95% CI: 1.61–4.34)], although the quality of the evidence is overall low.^175^ However, a 2020 Cochrane review of 14 randomized control trials (RCTs) including 1,563 women with endometriosis comparing laparoscopic ablation and/or excision with any other laparoscopic or robotic intervention, medical or holistic treatment, or diagnostic laparoscopy alone, found insufficient data to determine if laparoscopic surgery reduces overall pain with endometriosis at 6 and 12 months.^169^

##### 2.6.2.3 Fertility

Research on fertility outcomes after endometriosis surgery is limited. Earlier analysis suggested an improvement in live birth rates and pregnancy rates after surgery for mostly superficial/early‐stage disease.^170^, ^173^ However, more recent studies suggest it remains unclear if operative laparoscopy improves clinical pregnancy or live birth rates,^174^, ^175^ but may improve viable intrauterine pregnancy rates compared to diagnostic laparoscopy alone [OR 1.89 (95% CI: 1.25–2.83); 3 RCTs of 528 patients].^169^ There are no RCTs on live birth rates.^169^ Further controversy exists for patients undergoing in vitro fertilization (IVF). As of this publication, there are also no RCTs comparing reproductive outcomes after surgery in infertile women with deep endometriosis undergoing IVF.^176^, ^177^ However, a systematic review of a total of four studies on this topic found a pregnancy rate per patient of 1.84 (95% CI: 1.28–2.64), pregnancy rate per cycle of 1.84 (95% CI: 1.26–2.70), and live birth rate per patient of 2.22 (95% CI: 1.42–3.46) times more for patients who underwent surgery.^177^ In the case of ovarian endometriomas greater than 3–4 cm, surgical excision of the ovarian cyst capsule is associated with improved spontaneous pregnancy rates in women with previous subfertility, pain relief, and recurrence rates compared to drainage and ablation of the cyst wall.^178^ Surgery, however, in some cases can cause adverse effects on fertility by decreasing ovarian reserve (in the case of ovarian cystectomy for endometriomas), potential delays in fertility treatments, and the development of adhesions.^178^ Given what is currently known, surgeons are often left to make clinical decisions that may improve or further impair fertility of their patients based on very limited data.

##### 2.6.2.4 Laparoscopic excision versus ablation

Surgical excision and ablation have been shown to improve pain outcomes.^179^ In 2021, Burks et al. performed a systematic review and meta‐analysis of excision versus ablation in those with minimal‐to‐mild endometriosis. Three RCTs were included of a total of 346 patients with follow‐up time ranging from 6 to 60 months post‐operatively. From this limited amount of data, no significant difference in mean visual analog scale (VAS) was seen between excision and ablation in terms of dysmenorrhea, dyspareunia, or dyschezia.^180^ A 2021 systematic review and meta‐analysis of two RCTs from 2001 and 2002 of patients with mild‐to‐moderate stage endometriosis concluded that laparoscopic excision of endometriosis is superior to ablation in all aspects of endometriosis‐associated pain including dysmenorrhea, dyspareunia, dyschezia, and chronic pelvic pain.^181^ The only study with longer term follow‐up by Healey in 2014 reported a double‐blind randomized trial of patients with non‐deeply infiltrating endometriosis followed over 5 years.^171^ This study found a reduction in VAS scores for both excision and ablation with a significantly greater reduction in dyspareunia for the excision group. Additionally, more women in the ablation group continued to receive medical therapy for endometriosis at 5 years.^171^ Of note, the same group reported the data from the same cohort at only 1 year which did not show any significant difference between excision versus ablation. Healey's studies also excluded deeply infiltrative endometriosis and to our knowledge, only one RCT reports conservative laparoscopic treatment versus colorectal resection of deep endometriosis infiltrating the rectum.^169^ Overall, there is insufficient evidence to determine if there is a difference between laparoscopic ablation or excision of endometriosis and only two RCTs of direct comparison currently exist.^169^ However, the long‐term study favors excision.

##### 2.6.2.5 Recurrence

Recurrence of endometriosis after surgery is variably defined as a return of pain, imaging findings of endometriosis, or the need for repeat surgery.^182^ Rates of recurrence after surgery were 21.5% at 2 years and 40%–50% at 5 years.^183^ Reoperation is common, occurring in over 50% of patients and 27% undergo three or more surgeries.^184^ The pathophysiology of recurrence is not clear, but may be due to incompletely excised lesions, residual microscopic disease, or growth of new implants and is higher among those with advanced disease or clinical severity, younger age, and those who choose ovarian or uterine conservation.^185^

Preoperative planning is essential in the surgical management of endometriosis. Up to 37% of patients with deep infiltrating endometriosis (DIE) have intestinal involvement and incomplete excision may be associated with higher rates of recurrence.^186^, ^187^ As discussed above, a model combining patient history, symptoms, and imaging with ultrasound and/or MRI predicts rASRM stages III/IV.^114^ Conservative surgery with ovarian and/or uterine conservation is often performed to maintain reproductive potential, however, is also associated with higher rates of recurrence of symptoms and the need for repeat surgery.^188^ Post‐operative hormonal suppression with progesterone‐containing medical therapy, androgenic agents, and GnRH analogs may decrease recurrence by suppressing ovulation, retrograde menstruation, and proliferation of endometriotic implants stimulated by estrogen from retained ovaries. An initial 2004 Cochrane Review of 12 RCTs comparing post‐operative hormonal treatment for endometriosis to no therapy showed no benefit in recurrence rates, pain scales, or pregnancy rates at 3–6 months.^189^ Additionally, side effects of certain classes of hormonal suppression medications (e.g., GnRH analogs) may limit their long‐term use. More recent studies, however, of long‐term hormonal suppression past 6 months with combined estrogen‐progestin oral contraceptive pills (OCPs) or progestin‐only pills, and the levonorgestrel intrauterine system support use to prevent recurrence of endometriomas and dysmenorrhea.^190^ The literature on prevention of dyspareunia or non‐cyclic pelvic pain, however, is limited.^190^ As deep dyspareunia, non‐cyclic pelvic pain, and adhesions are more likely in patients with DIE, it remains unclear if long‐term OCPs or progestins prevent recurrence.

##### 2.6.2.6 Areas for improvement

The lack of clear evidence of risks and benefits, comparisons of different treatment modalities (e.g., IVF/IUI), and required expertise of surgeons complicates the decision to intervene with surgery. Comparisons in RCTs of surgical management among different subtypes (superficial, ovarian endometriomas, and deep endometriosis), using a variety of techniques and modalities (i.e., robotics, argon plasma, helium gas, laser, etc.) are also needed as well as comparisons of surgery overall to more holistic integrative options. Additionally, improved preoperative biomarkers or imaging that can predict response to surgery versus medical or integrative management, likelihood of disease recurrence, and potential effects on fertility would greatly improve clinical care and future advances in precision medicine may play a role. Unfortunately, as with most areas of clinical care improvement in surgical care for marginalized communities deserves further attention and intervention. With regard to endometriosis surgery, a retrospective cohort study of American College of Surgeons National Surgical Quality Improvement Program data from 2010 to 2018 found higher perioperative complication rates among patients who are American Indian or Alaska Native (adjusted OR (aOR) 2.34, 95% CI: 1.32–4.17), Native Hawaiian or Pacific Islander (aOR 2.08, 95% CI: 1.28–3.37), Black or African American (aOR 1.71, 95% CI: 1.39–2.10), and Hispanic (aOR 1.31, 95% CI: 1.06–1.64) compared to patients who are White.^191^

### 2.7 Endometriosis across the lifespan

#### 2.7.1 Adolescents

Prevalence of endometriosis in adolescents has been estimated between 19 and 73% of those presenting with severe dysmenorrhea and chronic pelvic pain, with identification of disease at surgery or by imaging.^192^ Recent guidelines favor diagnosis by history of symptoms, age at menarche, obstructive genital malformations, and family history of endometriosis, and by pelvic exam and transvaginal ultrasound (if tolerable), and laparoscopy.^117^, ^192^ Treatment for pain associated with suspected endometriosis includes NSAIDs, hormonal contraceptives, or progestogens as first‐line therapies, with GnRH analogs considered if symptoms persist along with hormonal add‐back therapy as adolescent bone density may not have yet reached its maximum. Surgery is another option for treatment (in addition to diagnosis). Patient education about the disease and a discussion about possibly undergoing oocyte cryopreservation for fertility preservation is recommended, although long‐term safety in adolescents with endometriosis for this procedure is unknown.^117^ Moreover, the cost of oocyte cryopreservation likely would be anticipated to exclude socioeconomically disadvantaged populations. Unfortunately, endometriosis is commonly not considered among possible causes of debilitating symptoms among adolescents because health care providers and family members and friends may not be familiar with the disorder and how to diagnose or treat it. Moreover, young women are often assumed to be somaticizing symptoms, which lengthens the path to diagnosis and treatment (see Disparities section below). Major efforts are underway to increase awareness of endometriosis among the lay population (e.g., the recent World Health Organization Endometriosis Fact Sheet (https://www.who.int/news‐room/fact‐sheets/detail/endometriosis)) and among health care providers.

#### 2.7.2 Post‐menopause

At the other end of the reproductive lifespan, endometriosis can still persist or rarely develop de novo. While endometriosis is an estrogen‐dependent disorder and most affected patients have remission of their symptoms post‐menopause, the disease also can synthesize E_2_ and be auto‐stimulating in the absence of ovarian function or the ovaries, per se. Evaluation depends on symptoms and usually is by imaging and history, and aromatase inhibitors have been used with variable outcomes.^117^ Other causes of pelvic pain or ovarian or pelvic masses warrant thorough evaluation, as the risk of malignancy independent of endometriosis history is higher with age. Surgical evaluation and treatment are another option, if risks outweigh the benefits.

### 2.8 Disparities and health equity

#### 2.8.1 Diagnosis

Historically, endometriosis has been classified as a condition characterized by its prevalence among individuals of certain genders, class, and race/ethnicity.^193^ Dr. J. Meigs initially proposed an etiologic role of contraception use and delayed childbearing for endometriosis.^194^ Therefore, middle‐income, White, cis‐gendered women of reproductive age have for a long time been portrayed as the focus of this disease, which leaves out many marginalized communities quite affected by the disease.^15^, ^195^ Several studies subsequently support a higher prevalence in White and Asian cis‐gendered women; however, many of these studies were methodologically flawed—for example using incomparable exposure populations (insured White women compared to uninsured or underinsured Black women or a small proportion of non‐White women).^195^ Additionally, more recent studies by study design rather outline who is offered a laparoscopy for chronic pelvic pain and/or infertility than a true endometriosis prevalence.^15^ In a study conducted by Chatman as early as 1975, specifically in 190 Black women, previously clinically diagnosed and treated for pelvic inflammatory disease found endometriosis present in as high as 21%, suggesting misdiagnosis of Black women with more acute causes of chronic pelvic pain.^196^ However, the long‐standing narrative of rare disease in non‐White and Asian women has had an impact on medical education of providers and continues to be present in the medical literature.^195^

As noted above, the true prevalence of endometriosis is difficult to access as definitive diagnosis requires surgical evaluation and access to surgery, and access is not equal across populations, in particular for minimally invasive laparoscopic/robotic surgery (MIS). Racial/ethnic and socioeconomic disparities in access to gynecologic surgery are known, with historically excluded minorities and lower income communities groups receiving less MIS surgery and living in areas with less MIS surgeons.^112^, ^113^ This disparity in access to MIS surgery suggests a likely underdiagnosis of endometriosis in these communities.

Additionally, few studies include analyses of clinical characteristics in other racial and ethnic groups such as indigenous Americans, non‐European/American/Asian populations, transgender men, and adolescents. As such, health care providers are often unfamiliar with the complete and heterogeneous clinical presentation of endometriosis. This “leaving out” has led to more delayed diagnoses among already disenfranchised communities, transgender men, and adolescents. A systematic review of 18 randomized control trials and observational studies found that Black and Hispanic women were ~50% less likely to be diagnosed with endometriosis as opposed to white women (OR: 0.49, 95% CI: 0.29–0.83, OR: 0.46, 95% CI: 0.14–1.50, respectively), although not statistically significant for Hispanic women.^15^ Furthermore, Asian women were more likely to be diagnosed in comparison with white women (OR: 1.63, 95% CI: 1.03–2.58).^15^ Among transgender men, some studies suggest that this group may have a higher prevalence of endometriosis than cis‐gender women^191^; however, studies that include this population are extremely limited. This indicates that there is much to study as the profile of this disease is changing and we still do not know if differences observed are related to diagnostic disparities or true variations based on ethnic groups. Furthermore, future research must include diverse populations including various racial/ethnic, transgender and gender non‐conforming, and adolescent populations and consider the impact of structural racism, gender inequity, and implicit bias on study design and interpretation.

Even in those with more classic symptoms of endometriosis, diagnoses may be delayed in underserved communities. One of the most common symptoms of endometriosis, pelvic pain can be an important symptom that can lead to the diagnosis of the disease. However, historical manifestations of racism against Black people may also contribute to the disproportionate rates of endometriosis diagnoses. Studies demonstrate that throughout history, medical institutions have furthered stereotypes that Black people are less susceptible to pain.^197^ Furthermore, across many different types of pain experienced by patients, Black and Brown patients are often undertreated and underdiagnosed in comparison with non‐Hispanic white patients.^195^, ^197^ Hispanic patients who have endometriosis have identified that their dysmenorrhea and high pain are particularly severe, and negatively impact their quality of life.^198^ Therefore, these implicit biases that providers impart to their patients and junior‐level trainees have detrimental impacts on patient care. Thus, it is particularly important to understand how to address implicit biases in health care as we attempt to understand how to better attend to the needs of marginalized communities affected by endometriosis. If true differences do exist among racial/ethnic groups in the United States, further exploration into the structural, societal, and environmental exposures is needed in addition to evaluation of any genetic variation among ethnic groups.

#### 2.8.2 Treatment

With regard to quality of care, even when historically excluded communities attempt to access care, other difficulties can negatively impact the quality of care that they receive. Overall, surgical diagnosis of endometriosis takes about seven years to occur, and this time is often prolonged in areas that have limited resources.^199^ Considering that marginalized communities often live in low‐resource areas, they may not be able to receive proper and timely care for their diagnoses. Additionally, patients who also utilize public insurance programs like Medicaid and Medicare in the U.S. are three times less likely to obtain medical services like radiology and laparoscopy and are more likely to be prescribed opioid and narcotic medications in comparison with those with private insurance.^200^ This suggests that people who are of low socioeconomic status receive differential care that is not as robust or comprehensive as their higher socioeconomic counterparts. Furthermore, patients who identified as Black (adjusted OR (aOR) 1.71 95% CI: 1.39–2.10), Hispanic (aOR 1.31 95% CI: 1.06–1.64), Pacific Islander, or American Indian (aOR 2.08, 95% CI: 1.28–3.37), or American Indian or Alaska Native (aOR 2.34, 95% CI: 1.32–4.17) were more likely to experience elevated surgical complications related to endometriosis.^191^ For transgender individuals, there is a paucity of data on pain characterization and few studies evaluate the barriers faced when accessing health care,^201^ so there could be other ways in which endometriosis continues to be undertreated within the community. While innovative treatment options for endometriosis are still being identified, it is essential to ensure there is equity in the access to medical treatments and highly skilled surgeons, improvement in environmental exposures, and greater patient and provider education for all populations to ensure that health care is being properly addressed.

## 3 SUMMARY AND EYE TO THE FUTURE

**FIGURE 7:**
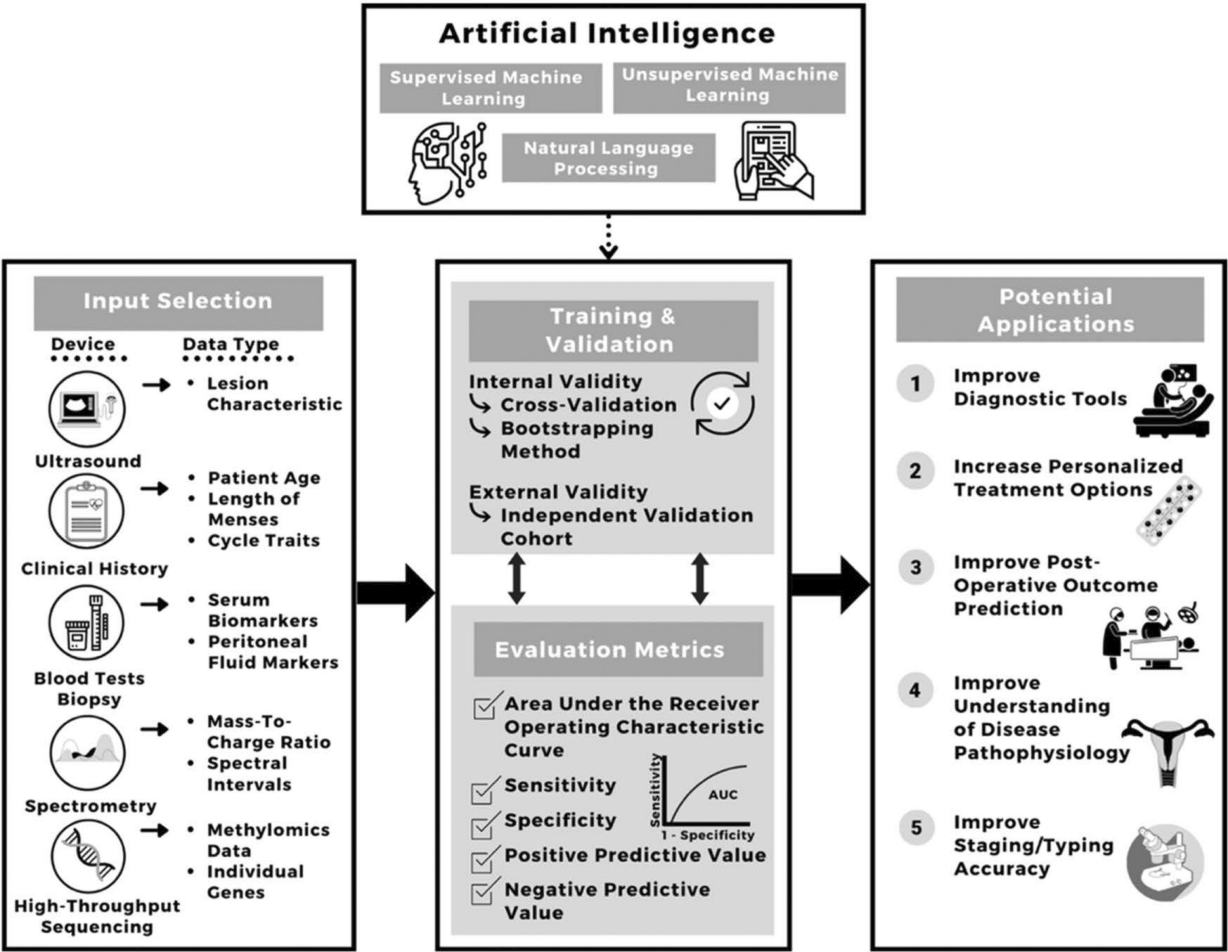
Potential indications for artificial intelligence applications in endometriosis. Artificial intelligence, combining supervised and unsupervised machine learning and natural language processing, with foundational input selections, training, and validation, is proposed to lead to improved diagnostics, therapeutics, post‐operative outcome predictions, disease pathophysiology understanding, and phenotyping of endometriosis. From Ref. [202]: Sivajohan B, Elgendi M, Menon C, Allaire C, Yong P, Bedaiwy MA. Clinical use of artificial intelligence in endometriosis: a scoping review. NPJ Digit Med. 2022;5(1):1–17. 10.1038/s41746‐022‐00638‐1, with permission.

Endometriosis is an extraordinarily complex disease that has significant gaps in expediency and accuracy of diagnosis, medical and surgical therapies that are variably effective for pain and infertility for the *individual*, and huge disparities among populations. Recent advances in big data and informatics allow for integrative approaches to derive insight into diseases while taking into account the complexity and individual variability in disease via precision medicine, and thus are well positioned to be pursued in endometriosis research. Molecular profiling allows enhanced understanding of disease mechanisms at a cellular level and developing novel therapeutic and diagnostic strategies. Electronic medical records (EMR) are an emerging underutilized data source with extensive longitudinal clinical information including diagnoses, medications, and labs. These data have yet to be fully utilized to study disease heterogeneity in endometriosis, with most prior studies only focusing on individual data realms (e.g., clinical trials, billing, diagnosis), and even fewer studies in diverse patient populations. Both clinical and molecular data‐driven approaches can be applied to derive new insights and hypotheses into disease heterogeneity, particularly relevant to endometriosis. Emergence of modeling methods also provides opportunities for phenotyping and predictive modeling of disease onset on longitudinal clinical data allowing for more precise deciphering of disease mechanisms underlying heterogeneous clinical manifestation, as well as improvements in diagnosis. Identification of clinical features will allow the generation of hypotheses that can inform future studies to verify specific pathogenesis and phenotypes that contribute to different disease and response groups. Furthermore, implementation of predictive models on clinical data can help guide patients and clinicians to consider disease risk and preventative measures. By making use of rich clinical and molecular data from diverse populations clinicians, together with basic and computational scientists can work together to advance endometriosis research and guide clinical care through improved patient stratification and ultimately personalization of risk identification or treatment approaches in endometriosis enabling precision medicine for all. A recent scoping review has highlighted artificial intelligence and machine learning algorithms to integrate complex metadata, omics data, diagnostic approaches, and therapeutic targets to improve endometriosis patient diagnosis, disease phenotyping, personalized therapies, prognostic indicators of responses to treatment, and risk of recurrence (Figure 7).^202^ In this space, the future is now, and endometriosis warrants being in the front of the line to move this enigmatic disease forward for the benefit of those affected. We anticipate multidisciplinary approaches and leveraging clinical data across diverse patient cohorts will further inform endometriosis disease mechanisms underlying the known heterogeneous clinical manifestations and improve patient stratification and personalized clinical approaches to therapies. Moreover, it is imperative that research studies consider and involve diverse populations, including those from racial and ethnic minorities and transgender individuals, so that factors that contribute to the disease can be fully understood, and clinical and biomedical advances will benefit everyone—not just select groups.^203^

## AUTHOR CONTRIBUTIONS

Linda C. Giudice conceived and structured the review. All authors were involved in drafting different sections of the manuscript, editing further, and literature citations. Linda C. Giudice wrote the sections on pathogenesis and pathophysiology, diagnosis, biomarkers, and medical therapies. Marina Sirota wrote sections on drug repurposing, AI, and precision medicine. Tomiko T. Oskotsky wrote sections on epidemiology and mHealth and synthesized the Zotero reference list. Simileoluwa Falako and Jessica Opoku‐Anane wrote sections on health disparities, and Jessica Opoku‐Anane wrote a section on surgical therapies.

## DISCLOSURES

Linda C. Giudice is a consultant to Myovant Sciences, Celmatix, NextGen Jane, and Gesynta. Jessica Opoku‐Anane is a consultant to Myovant Sciences, Boston Scientific, and Abbvie. Other authors declare no conflicts of interest.

## Data Availability

Data sharing is not applicable to this article as no datasets were generated or analyzed during the current study.
